# Structural basis of IgLON5 autoantibody recognition in autoimmune encephalitis

**DOI:** 10.1038/s41467-026-77812-6

**Published:** 2026-09-11

**Authors:** Angelique Roux, Rachel Schelling, David Vinyals-Sales, Lidia Sabater, Rahel R. Winiger, Ilayda Senyuz, Alison Lin, Amandine Mathias, Renaud Du Pasquier, Carles Gaig, Josep Dalmau, Mathilde Foglierini, Laurent Perez

**Affiliations:** 1 Department of Medicine, Service of Immunology and Allergy, Lausanne University Hospital and University of Lausanne, Lausanne, Switzerland; 2 Centre for Human Immunology, Lausanne, Switzerland; 3 Neuroimmunology Program, Fundació de Recerca Clínic Barcelona, Institut d’Investigacions Biomèdiques August Pi i Sunyer; and Caixa Research Institute (CRI), Universitat de Barcelona, Barcelona, Spain. Centro de Investigación Biomédica en Red de Enfermedades Raras (CIBERER), Madrid, Spain; 4 Laboratory of Neuroimmunology, Neuroscience Research Centre, Department of Clinical Neurosciences, Lausanne University Hospital and University of Lausanne, Lausanne, Switzerland; 5 Multidisciplinary Sleep Disorders Unit, Hospital Clinic de Barcelona, Barcelona, Spain. Neurology Service, Hospital Clinic of Barcelona. Institut d’Investigacions Biomèdiques August Pi i Sunyer (IDIBAPS), Barcelona, Spain; 6 CaixaResearch Institute, Barcelona, Spain; 7 Department of Neurology, Perelman School of Medicine, University of Pennsylvania, Philadelphia, PA USA

**Keywords:** Autoimmunity, Cryoelectron microscopy, Encephalopathy, Neurodegeneration, Molecular medicine

## Abstract

Anti-IgLON5 disease is a rare neuro-immunological disorder characterized by autoantibodies targeting the neuronal adhesion molecule IgLON5, as well as profound brain dysfunction and tau pathology. Despite its severe clinical manifestations, the molecular basis of antibody recognition and its contribution to disease pathogenesis is poorly understood. Here, we characterize the B cell receptor repertoire of a patient with anti-IgLON5 disease, revealing marked diversity and no evidence of dominant clonal expansion. We isolate a human monoclonal IgG4 antibody that binds IgLON5 with high affinity and determine the structure of its Fab in complex with IgLON5 using cryo–electron microscopy. Biochemical and structural analyses show that antibody binding preserves IgLON5 adhesive interfaces while remaining compatible with clustering of IgLON5 on the cell surface. Analysis of the germline-reverted precursor suggests that IgLON5 recognition is already present before affinity maturation and is strengthened by somatic mutations that stabilize antigen binding. These findings provide mechanistic insight into autoantibody recognition of neuronal surface proteins and establish a framework for understanding antibody-mediated neurodegeneration in anti-IgLON5 disease.

## Abstract (web summary)

Anti-IgLON5 disease is a rare neuro-immunological disorder characterized by the production of autoantibodies against the cell adhesion molecule, IgLON5. Here, the authors examine the B cell receptor repertoire of an individual with this condition and present a cryo–electron microscopy structure of a patient-derived antibody clone in complex with IgLON5.

## Introduction

IgLON proteins comprise a family of immunoglobulin-like cell adhesion molecules (CAMs) predominantly localized to neuronal membranes, where they contribute to the organization and maintenance of nervous system architecture^1,2^. These proteins play key roles in regulating neuronal arborization, supporting the formation and maintenance of excitatory synapses, and modulating neuronal connectivity and network stability^3–6^. The protein family comprises five members: opioid-binding protein/cell adhesion molecule-like (OPCML/IgLON1), neurotrimin (NTM/IgLON2), limbic system-associated membrane protein (LSAMP/IgLON3), neuronal growth regulator 1 (NEGR1/IgLON4), and IgLON5^7^. They all share a high degree of structural similarity with a length of about 340 amino acids divided in three extracellular immunoglobulin (Ig)-like domains of the C2 type followed by a short stalk and a glycosylphosphatidylinositol (GPI)-anchor^8,9^. All IgLON family members assemble into homo- and heteromeric complexes, engaging in both *cis* and *trans* interactions to mediate cell–cell adhesion and regulate neuronal connectivity^10,11^. IgLON dimerization is mediated by the first Ig domain, with the two protomers associating in a “head-to-head” configuration^8^. These *trans* interactions (across opposing membranes) generate a V-shaped complex compatible with synaptic cleft dimensions^12^ and likely contribute to the organization of chemical synapses^13^. Moreover, a study in *C. elegans* provided evidence that trans-synaptic IgLON interactions can organize neurochemical synapses and suggested that IgLONs may also interact with ionotropic receptors in the mammalian nervous system^14^.

Among the IgLON family members, IgLON5 has attracted particular attention following the identification of anti-IgLON5 disease, a rare but severe neurological disorder characterized by the presence of IgG autoantibodies targeting IgLON5 in both cerebrospinal fluid and serum^15^. Clinically, patients present with a heterogeneous constellation of symptoms, including rapid eye movement (REM) and non-REM parasomnias, sleep apnea, bulbar dysfunction, gait instability, and abnormal movements, mainly facial dyskinesia and chorea^16,17^. The disorder frequently evolves into a neurodegenerative state marked by neuronal accumulation of hyperphosphorylated tau across multiple regions of the central nervous system with preference in the tegmentum of the brainstem, hypothalamus, and spinal cord^18,19^. Notably, the disease exhibits a strong genetic association with specific human leukocyte antigen (HLA) haplotypes, particularly HLA-DRB1*10:01 and DQB1*05:01, supporting a role for genetic predisposition in disease susceptibility and highlighting a link between autoimmune mechanisms and neurodegeneration^20^. Similarly to other autoimmune encephalitis syndromes, such as LGI1 (leucine-rich glioma-inactivated 1) and CASPR2 (contactin-associated protein-like 2) diseases, autoantibodies predominantly belong to the IgG4 subclass, although IgG1 antibodies have also been reported^21,22^. IgG4 responses are typically associated with chronic antigen exposure and are characterized by limited effector functions, including a reduced capacity to activate complement and engage Fc receptors^23^, suggesting that disease mechanisms may extend beyond classical antibody-mediated cytotoxicity. Despite the severity and progressive nature of the disorder, the fundamental immunological and molecular pathways driving disease pathogenesis remain poorly defined, and current therapeutic approaches have demonstrated only modest efficacy^24,25^.

Recent studies have demonstrated that polyclonal IgG isolated from patient sera can disrupt trans-synaptic interactions and induce a sustained reduction of IgLON5 clusters at the neuronal surface, thereby underscoring the critical role of IgLON5 in maintaining neuronal connectivity^26,27^. Furthermore, exposure of neurons to anti-IgLON5 antibodies results in neurodegenerative alterations, including membrane blebbing and neuritic fragmentation probably leading to increased tau hyperphosphorylation and ultimately neuronal cell death, as shown in autopsy studies^28,29^. These effects are thought to arise from antibody-induced neuronal hyperactivity, which promotes somatodendritic tau missorting and aggregation^30^. Interestingly, distinct mechanisms of action have been proposed depending on the immunoglobulin subclass involved: IgG1 antibodies have been shown to induce irreversible internalization of IgLON5 clusters, whereas IgG4 antibodies are more likely to interfere with protein–protein interactions^27^. Clinically and neuropathologically, the disease represents a unique form of tauopathy that differs from classical primary tauopathies such as Alzheimer’s disease, progressive supranuclear palsy (PSP), and corticobasal degeneration (CBD). IgLON5 tauopathy is characterized by the accumulation of both 3 R and 4 R tau isoforms, yet displays a distinct anatomical distribution, differing from the cortical predominance observed in Alzheimer’s disease and the basal ganglia–frontal involvement typical of PSP and CBD^19,29,31^. Collectively, these findings support a model in which tau deposition in IgLON5 disease arises secondary to chronic antibody-mediated synaptic dysfunction, rather than reflecting a primary tau-driven neurodegenerative process.

Most studies investigating IgLON5 autoimmunity and antibody binding have relied on polyclonal IgG purified from patient sera. While informative, such approaches preclude the precise dissection of individual antibody specificities and limit detailed structural and mechanistic characterization of IgLON5 recognition. In this study, we dissected the B cell repertoire of a patient with anti-IgLON5 disease, revealing a diverse, polyclonal IgG4-skewed response. Despite the lack of dominant clonal expansion, we isolated a high-affinity monoclonal antibody, suggesting that pathogenic activity may be driven by individual clones embedded within a broadly heterogeneous repertoire. Using single-particle cryo–electron microscopy (cryo-EM), we resolved the structure of the IgLON5–Fab complex at 3.7 Å resolution, revealing that the antibody targets a broad conformational epitope within the second Ig-like domain. Importantly, our data demonstrate that Fab binding does not interfere with IgLON5 homo- or heterodimerization, indicating that pathogenicity is unlikely to arise from direct disruption of adhesion interfaces. Instead, structural organization combined with in vitro assays supports a model in which bivalent antibody binding promotes higher-order clustering of IgLON5 homo- and heterodimers, thereby providing a structural basis for antibody-driven synaptic reorganization.

## Results

### Characterization of the B cell receptor repertoire in anti-IgLON5 disease

**Fig. 1:**
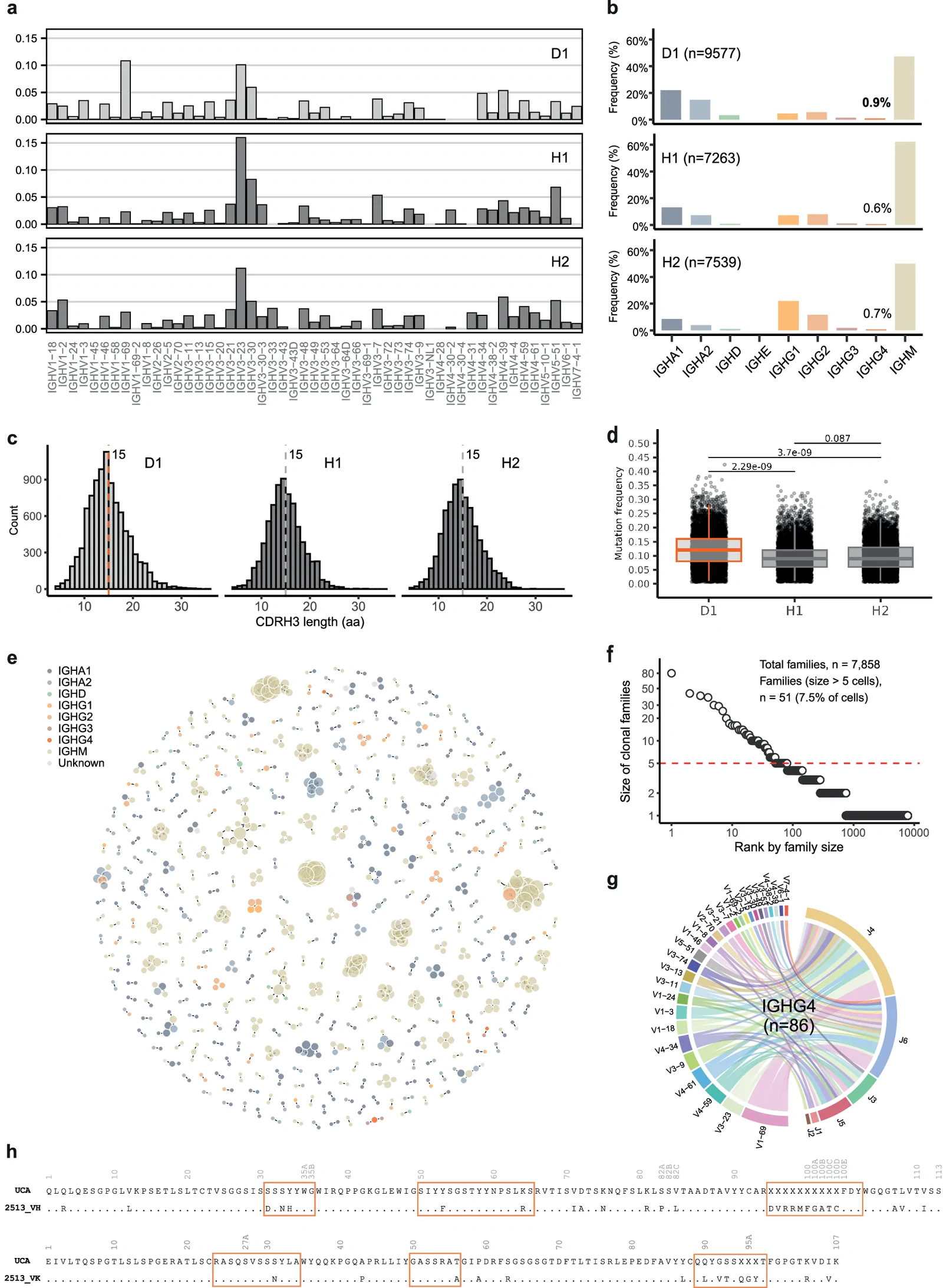
BCR repertoire profiling and clonal architecture of memory B cells in anti-IgLON5 disease. **a** Bar chart showing the frequency of VH gene usage among paired BCRs in memory B cell populations from anti-IgLON5 patient D1 and two healthy donors (H1 and H2). **b** Isotype frequency distribution in donors D1, H1, and H2. The total number of clonotypes per donor is indicated in parentheses. The frequency of IgG4 is annotated on the corresponding bar. **c** Histogram of CDRH3 length distribution for donors D1, H1 and H2. Dashed lines indicate median values. **d** Boxplot showing somatic mutation frequency (amino acid substitutions relative to V gene germline) across the three donors (*n* = 7178 for donor 1, *n* = 7019 for H1 and *n* = 7117 for H2). Boxes represent the interquartile range (IQR; 25th–75th percentile), with the central line indicating the median. Whiskers extend to 1.5× the IQR, and individual points beyond this range are plotted as outliers. Significant differences were assessed by Welch’s ANOVA (F (2, 14141) = 404, *p* < 0.0001), with post hoc two-sided Games–Howell testing revealing significant differences between D1 and H1 (*p* < 0.0001) and D1 and H2 (*p* < 0.0001), but not between H1 and H2 (*p* = 0.087). **e** Clonal network of CDRH3 sequences, colored by isotype. Clonal families were defined using the Change-O Immcantation toolkit incorporating both heavy and light chains of paired BCRs. Clonal family identifiers were subsequently used as V gene input for edit-distance-based clustering with clonalCluster() (scRepertoire). Node size is proportional to the square root of the number of identical sequences. **f** Waterfall plot showing the size distribution of all clonal families. A dashed red line delineates clonal families containing more than five cells from those containing five or fewer cells. **g** Chord diagram representing heavy chain V–J gene combinations among IgG4 clonotypes. **h** Sequence alignment of the IgLON5-specific monoclonal antibody mAb2513 with its inferred unmutated common ancestor (UCA). Top: heavy chain alignment, VH4-39 and JH4 (D gene region masked and represented as “X”). Bottom: light chain alignment, VK3-20 and JK3. Dots indicate identity with the UCA sequence. Residue positions are numbered according to the Kabat scheme. Red boxes highlight the three Kabat-defined complementarity-determining regions (CDRs).

To identify anti-IgLON5 monoclonal antibodies (mAbs), we sequenced the B cell receptor (BCR) repertoire from a 60-year-old male patient diagnosed with anti IgLON5 disease. Memory B cells isolated from peripheral blood mononuclear cells (PBMCs) of donor D1 were FACS sorted and subjected to single-cell BCR sequencing using the 10x Genomics platform. After filtering productive paired heavy and light chains sequences, we successfully reconstructed a set of 9577 immunoglobulin sequences from the donor, including 968 antigens specific (Supplementary Fig. 1). As a negative control, we used previously reported BCR repertoires from two healthy donors, matched for cell counts (H1 and H2)^32^. To assess BCR repertoire diversity across donors, we used coverage-based rarefaction analysis with Hill numbers. The estimated sample coverage ranged from ~0.10 to ~0.45 (Supplementary Fig. 2a) and despite incomplete sampling, D1 consistently exhibited the highest clonal diversity based on clonal richness (Hill q = 0) and Shannon diversity (Hill q = 1), compared to H1 and H2 (Supplementary Fig. 2b-c). This result, suggest a broader and more evenly distributed BCR repertoire in D1 and an absence of strong clonal expansion. The variable heavy (VH) gene usage was broadly similar across donors (Fig. 1a), except for a notable overrepresentation of VH1-69 in D1 relative to healthy controls. The light variable (VL) V gene usage was equally comparable, with no major differences in Kappa or Lambda gene frequencies across the three donors (Supplementary Fig. 3). Beyond V gene usage, isotype distribution was also largely conserved, with IgM⁺ clonotypes predominating across all donors and reaching 47.3% of the donor D1 repertoire. The IgA1 and IgA2 subtypes frequencies were modestly elevated in D1 (22% and 14.8%, respectively) relative to healthy donors (Fig. 1b). Surprisingly, only a slight increase in the IgG4 subtype was observed in the IgLON5 patient repertoire (D1: 0.9%; H1: 0.6%; H2: 0.7%). Consistent with the overall similarity in repertoire features, the length distribution of the third complementarity-determining region of the heavy chain (CDRH3) followed a normal distribution, with a median length of 15 residues across all donors (Fig. 1c). To assess the somatic mutation frequency, we first excluded clonotypes with a mutation frequency of zero, which likely included naive B cells, highly abundant in the donor D1 (25%) compared to healthy donors (H1: 3.4% and H2: 5.6%). Interestingly, the mutation frequency differed significantly across donors (*p* < 0.0001) and Post hoc Games–Howell testing showed that D1 had a significantly higher mean mutation frequency than both healthy donors (vs H1 and H2, *p* < 0.0001), whereas no significant difference was observed between them (H1 vs H2: *p* = 0.087). The mean mutation frequency in D1 was 0.121 ± 0.061, compared to 0.096 ± 0.050 in H1 and 0.098 ± 0.053 in H2 (Fig. 1d). Of note, this higher mutation frequency in D1 is consistent with previously reported increased frequency of somatic hypermutation in the context of autoimmune diseases due to chronic antigenic stimulation^33^. To define clonal relationships, we applied the Immcantation suite to reconstruct clonal families and represent the CDRH3 sequences as a clonal network (Fig. 1e). A total of 7858 clonal families were identified, illustrating a highly polyclonal BCR repertoire (Fig. 1f). The large clonal families (>5 cells) accounted for only 7.5% of total memory B cells and were predominantly of IgM isotype (68.6%). A smaller proportion of IgA clones (29.4%) and only rare IgG families were also detected. One prominent exception was a large clonal family of 80 IgM clonotypes utilizing VH1-69, consistent with the global overrepresentation of this gene segment in D1. As the disease is characterized by a predominance of IgG4 antibodies, we focused on this subclass and identified 86 paired sequences, including five antigen-specific sequences. The majority of IgG4 clones were singletons (one clonal family of size 3 and two of size 2) and heavy chain V–J gene combinations were highly heterogeneous (Fig. 1g). We then verified the specificity of the five antigen-specific clones by ELISA and confirmed the reactivity of one clone. Interestingly, the specific clone (mAb2513) utilized a VH derived from the germline gene IGHV4-39, which has been previously reported in several autoimmune conditions, including multiple sclerosis, where it has been associated with autoreactive B cell responses^34^. The inference of the unmutated common ancestor (UCA) for this clone (VH4-39/JH4 and VK3-20/JK3) revealed a somatic mutation frequency of 0.12, consistent with good affinity maturation of the clone (Fig. 1h).

### Biochemical characterization of mAb2513 and IgLON5 interaction

**Fig. 2:**
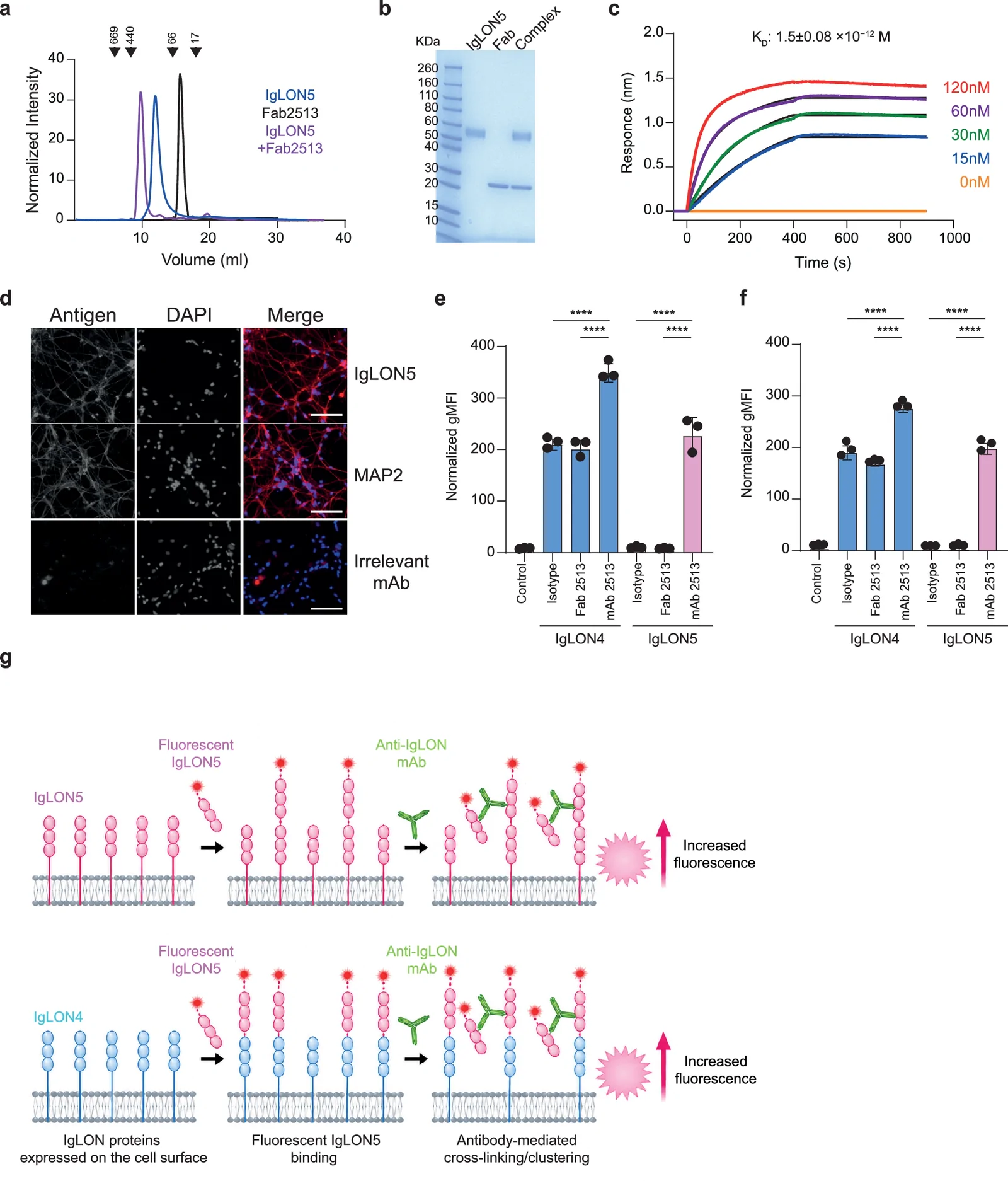
High-affinity interaction of mAb2513 with IgLON5 induces receptor clustering. **a** Size-exclusion chromatography profiles of IgLON5 (blue), Fab2513 (black), and the Fab–IgLON5 complex (violet). Arrowheads indicate the elution volumes of molecular weight markers: thyroglobulin (669 kDa), ferritin (440 kDa), BSA (66 kDa), and myoglobin (17 kDa). **b** SDS–PAGE analysis under reducing conditions of the corresponding peaks. IgLON5 migrates at 55 kDa due to glycosylation, while the reduced Fab fragment migrates at 25 kDa. SDS-PAGE image is a representative example of two independent experiments (*n* = 2). **c** Fab–IgLON5 interaction assessed by biolayer interferometry (BLI). The Fab was immobilized on FAB2G biosensor and exposed to IgLON5 at increasing concentrations (15–120 nM). Binding responses (nm shift) were recorded. The sensorgram shown is representative of two independent experiments (*n* = 2). **d** Immunostaining of human stem cell–derived neurons. IgG binding to MAP2-positive or IgLON5-expressing neurons is shown in red, and nuclei (DAPI) in blue. Scale bars, 50 μm. **e** Normalized geometric mean fluorescence intensity of IgLON4- or IgLON5-transfected SH-SY5Y cells incubated with fluorescent soluble IgLON5 in the presence of an irrelevant antibody, IgG1 mAb2513, or Fab2513. **f** Normalized geometric mean fluorescence intensity of IgLON4- or IgLON5-transfected SH-SY5Y cells incubated with fluorescent soluble IgLON5 in the presence of an irrelevant antibody (isotype), IgG4 mAb2513, or Fab2513. Control are mock transfected cells incubated with fluorescent soluble IgLON5. Data are presented as normalized geometric mean ± SD from three independent experiments (*n *= 3biological replicates). Significant differences were assessed by one-way ANOVA followed by a two-sided Tukey’s multiple-comparisons test, with ****P < 0.0001. For comparisons involving IgG1, the adjusted *P* values were as follows: IgLON4-mAb versus IgLON4-isotype, *P* = 7.68 × 10⁻⁷; IgLON4-mAb versus IgLON4-Fab, *P* = 3.57 × 10⁻⁷; IgLON5-mAb versus IgLON5-isotype, *P* = 1.69 × 10⁻⁹; and IgLON5-mAb versus IgLON5-Fab, *P* = 1.55 × 10⁻⁹. For comparisons involving IgG4, the adjusted *P* values were: IgLON4-mAb versus IgLON4-isotype, *P* = 1.27 × 10⁻⁸; IgLON4-mAb versus IgLON4-Fab, *P* = 5.69 × 10⁻¹⁰; IgLON5-mAb versus IgLON5-isotype, *P* = 1.01 × 10⁻¹³; and IgLON5-mAb versus IgLON5-Fab, *P* = 1.04 × 10⁻¹³. **g** Schematic representation of the potential receptor clustering induced by mAb2513.

To validate the reactivity of the VH4-39 BCR, we first generated a construct encoding the extracellular domain of human IgLON5 truncated at the ω-site (residue 316) to prevent glycosylphosphatidylinositol anchoring. We also added a Fc domain at the C-terminus, to ease protein secretion and purification. The protein was produced by transient transfection of Expi293 cells, purified by Protein A resin, and subsequently papain cleaved to remove the Fc tag. We next evaluated complex formation in solution using size-exclusion chromatography (SEC), by incubating tag-free IgLON5 with Fab2513 for 1 h at 4 °C. Separation of the constructs showed that IgLON5 forms a dimer, as expected^12^. In contrast, IgLON5 in complex with Fab2513 eluted as a high–molecular weight complex of ~240 kDa, consistent with two Fabs bound to the IgLON5 dimer (Fig. 2a, b). To confirm this finding and determine the affinity of Fab2513 to IgLON5, we used biolayer interferometry (BLI). We immobilized Fab2513 at 100 nM on biosensor tips and probed for interaction with a solution of IgLON5 at varying concentrations (Fig. 2c). We detected a high affinity interaction between Fab2513 and IgLON5, with a calculated *K*_D_ value of 1.5 ± 0.08 × 10^−12^ M. To assess potential cross-reactivity of the mAb2513, we tested its binding on the other IgLON family members by ELISA. We observed a strong reactivity for IgLON5, but no binding could be detected for the other protein members, highlighting the mAb high specificity (Supplementary Fig. 4). Given that purified IgG from patient sera have been shown to bind neuronal cell surfaces and induce IgLON5 clustering and internalization^26,30^, we investigated the cellular binding of mAb2513 on human induced pluripotent stem cell (hiPSC)-derived neurons^35^. We observed signal on cell bodies and dendrites (MAP2 + ) of neurons, while no signal was observed with an irrelevant antibody (palivizumab) confirming the mAb biological specificity (Fig. 2d). To investigate the effect of the antibody on IgLON organization at the cell surface, we used SH-SY5Y neuroblastoma cells transfected with either IgLON4 or IgLON5 and assessed their interaction with biotinylated recombinant IgLON5. The transfected cells were analysed by flow cytometry in the presence or absence of mAb2513 expressed as an IgG1, and we monitored geometric mean fluorescence intensity (gMFI) associated to the cell surface using fluorescent streptavidin. We observed a clear association of fluorescent biotinylated IgLON5 with IgLON4-transfected cells, whereas its association with IgLON5-transfected cells was minimal under these experimental conditions, as previously reported^12,26^ (Fig. 2e and Supplementary Fig. 5). Interestingly, in the presence of mAb2513 the fluorescence increased for both IgLON4 and IgLON5, (Fig. 2e and Supplementary Fig. 5). In contrast, no fluorescence increased was observed in the presence of an irrelevant human mAb (palivizumab), nor in the presence of Fab2513. This result demonstrates that mAb2513 can bind IgLON5 homo- and heterodimers in *trans* and may thereby induce clustering on neuronal cell surface. Since mAb2513 was originally isolated as an IgG4 antibody, an isotype known to undergo Fab-arm exchange^36,37^, we next repeated the same experiment using the original antibody isotype. Similar to its IgG1 counterpart, IgG4 mAb2513 increased the association of fluorescent biotinylated IgLON5 with IgLON4- and IgLON5-transfected cells, (Fig. 2f and Supplementary Fig. 5). This result indicates that mAb2513 retains trans-binding activity in its original isotype, although with reduced efficacy, possibly due to the presence of functionally monovalent antibodies generated by Fab-arm exchange (Fig. 2f and Supplementary Fig. 5). The proposed trans-crosslinking mechanism underlying both the IgG1 and IgG4 experiments is illustrated in the accompanying explanatory cartoon (Fig. 2g). In a polyclonal setting where multiple IgG4 antibodies coexist, Fab-arm exchange could potentially alter IgLON5 cross-linking in different ways, depending on the antigen specificity and epitope targeted by the exchanged Fab arms. Thus, Fab-arm exchange may reduce the effective bivalency of an individual anti-IgLON5 antibody but could also generate bispecific antibodies with distinct binding geometries and functional consequences^37^.

### Cryo-EM structure of the IgLON5 dimer in complex with Fab2513

**Fig. 3:**
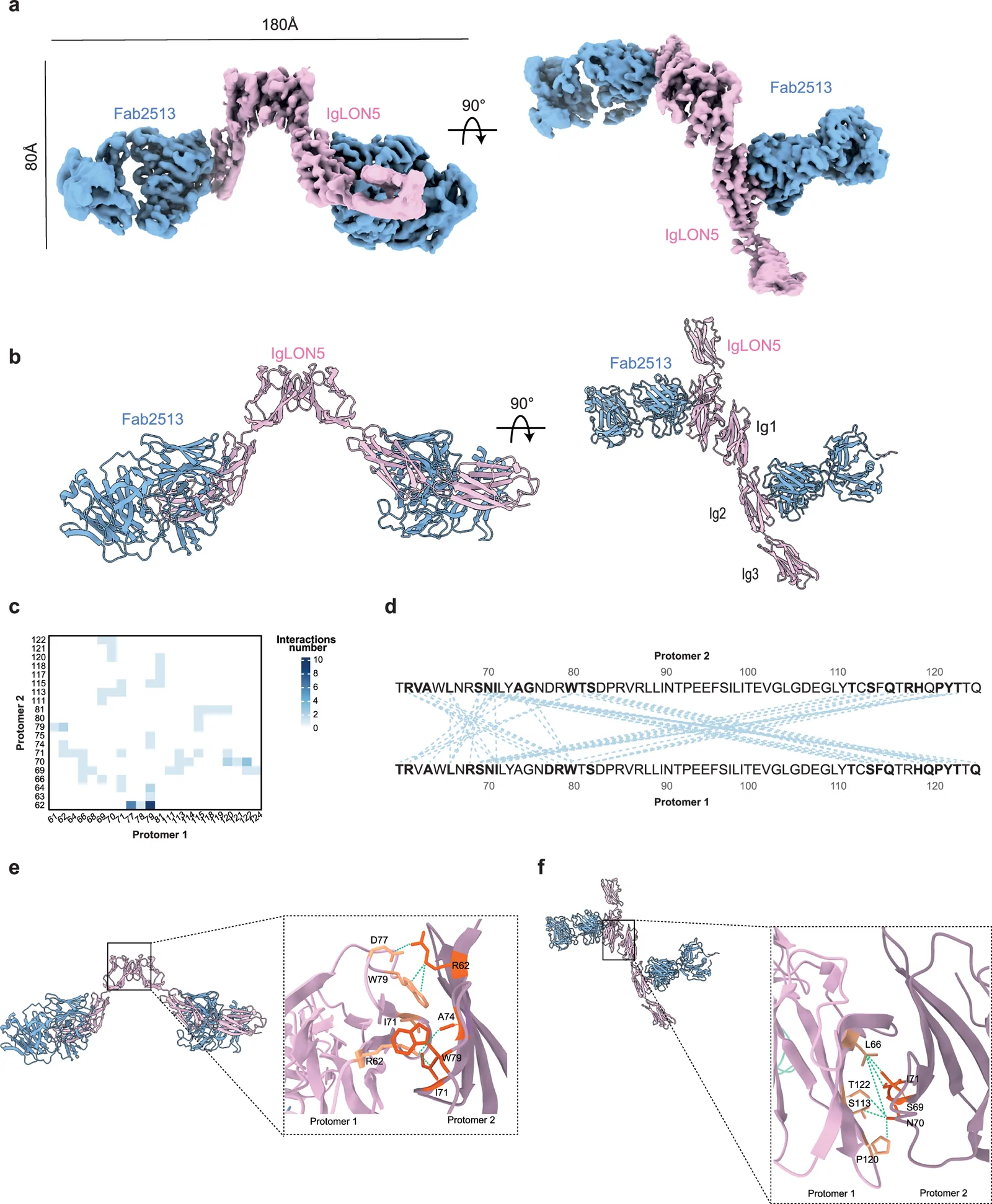
Structural characterization of the IgLON5 dimer bound by Fab2513. **a** Side and top views of the cryo-EM density map of two Fab2513 molecules in complex with the IgLON5 dimer, with IgLON5 shown in pink and Fab2513 shown in blue. The overall dimensions of the complex are indicated. **b** Atomic model of the complex shown in cartoon representation, with the three Ig domains labeled. **c** Heatmap showing the interactions between the two Ig1 domains of the IgLON5 dimer. The number of interactions is color-coded using a blue gradient. **d** 2D representation and sequence of the Ig1 domain for the protomer 1 and 2. Dashed lines indicate hydrogen bonds and salt-bridge interactions. The amino acids involved in the interaction are in bold. **e** Close-up view of the interactions involving arginine 62 (R62) and tryptophan 79 (W79). Protomer 1 is shown in pink, with highlighted residues in light orange, and protomer 2 is shown in violet, with highlighted residues in dark orange. **f** Close-up view of the interactions between the two protomers centered on leucine 66 (L66), isoleucine 71 (I71), and asparagine 70 (N70).

To define the molecular architecture of the complex, we performed single-particle cryo–electron microscopy and determined its structure at 3.7 Å resolution, estimated by Fourier shell correlation curves (FSC) (Table 1, Supplementary Figs. 6 and 7). The resulting cryo-EM map displayed higher-resolution features for the first two Ig-like domains (Ig1 and Ig2), whereas the third Ig-like domain (Ig3) was not resolved, likely due to pronounced interdomain flexibility, as previously reported^12^ (Fig. 3a). Structural analysis revealed that IgLON5 forms a homodimer within the complex, arranged in a head-to-head configuration through a common interface involving the first Ig-like domains, as previously described^12^. Structural comparison using pairwise structure alignment with TM-align^38^ showed that the Fab-bound IgLON5 dimer closely matches the previously published crystal structure of the IgLON5 dimer, with an RMSD of 1.4 Å, suggesting that the overall dimeric organization is preserved upon Fab binding (Supplementary Fig. 8). Interestingly, each Fab engages IgLON5 in an opposite orientation, with the epitopes positioned on opposing faces of the dimer (Fig. 3a, b). This spatial arrangement demonstrates that the antibody cannot bind bivalently to a single IgLON5 dimer. Instead, the two Fab arms could engage epitopes on adjacent dimers, thereby promoting receptor clustering. This result is consistent with the mAb2513-induced multimerization of IgLON5 on the surface of SH-SY5Y cells and provides a mechanistic explanation for the previously reported clustering observed with patient-derived IgG on murine neurons^27,30^. The IgLON5 dimerization is mediated by the two Ig1 domains, which bury approximately 780 Å² of surface area per monomer and orient the two monomers at a 90° angle relative to each other (Fig. 3b). The interface is stabilized by a dense and complementary combination of electrostatic, hydrogen-binding, and hydrophobic interactions distributed across the β-sheet faces and adjacent loop regions (Fig. 3c, d). A prominent electrostatic anchor is formed by a salt bridge between D77 of one protomer and R62 of the reciprocal protomer and reinforced by additional polar van der Waals and weak hydrogen contacts (CH–O/N), establishing a strong interdomain constraint (Fig. 3e). The R62 and W79 residues from one protomer form a central interaction hub, with W79 engaging R62 of the other protomer through CH–π interactions together with polar and apolar van der Waals contacts, while the R62 establishes multiple proximal contacts with I71, A74, and W79 creating a tightly packed core (Fig. 3e). The central portion of the interface is enriched in hydrophobic contacts. Reciprocal L66 and I71 form multiple proximal and apolar van der Waals interactions. These interactions generate a compact nonpolar interface that likely shields buried surfaces from solvent (Fig. 3f). Surrounding this hydrophobic core, an extended polar network contributes to specificity and orientation. Notably, N70 forms a hydrogen bond with S113 and engages in additional CH–O/N and apolar contacts with P120 and T122 (Fig. 3f). Reciprocally, S113 forms a hydrogen bond with S69, reinforcing the polar interaction network. Several additional proximal contacts involving S69, S81, Q115, and T122 with polar residues in domain I create an extended hydrogen-bond-compatible surface. Overall, the Ig1–Ig1 interface is characterized by a central aromatic–arginine interaction cluster, symmetric hydrophobic packing, and a peripheral web of hydrogen bonds and polar contacts. This combination of electrostatic anchoring, π-mediated interactions, and complementary van der Waals packing likely confers substantial rigidity to the Ig1 dimerization module. From a biological perspective, the rigid head-to-head dimerization, together with the observation that antibodies promote cross-linking of adjacent dimers rather than bivalent engagement of a single dimer, suggests that they primarily act by aberrantly clustering the receptor at the neuronal surface.

**Table 1:** Cryo-EM data collection, refinement and validation statistics

Data collection and preprocessing		
Microscope	FEI Titan Krios	
Voltage (kV)	300	
Electron exposure (e⁻/Å²/s)	17.34	
Defocus range (µm)	−1.2 to −2.4	
Magnification	81,000	
Pixel size (Å)	0.83	
Symmetry imposed	C1	
Initial particle images (no.)	3’169’956	
Final particle images (no.)	702’836	
	EMD-57080 EMD-57198	
Map resolution (Å)	3.7	3.5
FSC threshold	0.143	0.143
**Model building and Refinement**		
Model composition	PDB ID 29CP	PDB ID 29IN
Non-hydrogen atoms	7208	3979
Protein residues	1250	532
Chains	6	3
B factors (Å²)	73.92	57.95
R.m.s. deviations		
Bond lengths (Å)	0.004	0.003
Bond angles (°)	0.964	0.692
Validation		
MolProbity score	1.56	1.94
Clashscore	1.68	9.32
Rotamer outliers (%)	1.25	0.00
Ramachandran plot (%)		
Favored	89.18	93.16
Allowed	10.02	6.84
Outliers	0.81	0.00

### Characterization of Fab paratope and epitope

**Fig. 4:**
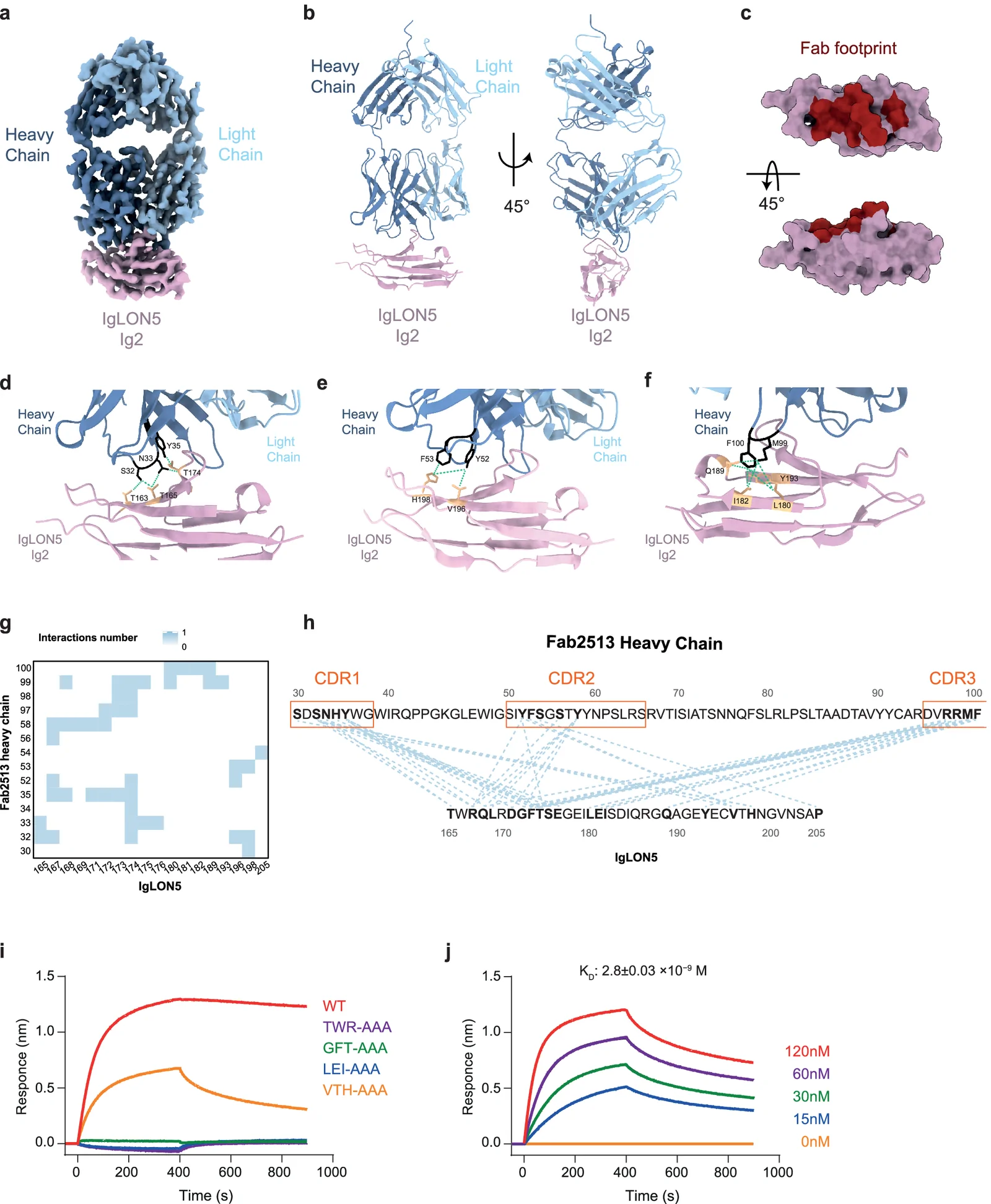
Detailed interaction of Fab2513 with the IgLON5 Ig2 domain. **a** Front view of the locally refined cryo-EM density map of Fab2513 bound to the Ig2 domain of IgLON5. The heavy chain is shown in dark blue, the light chain in light blue, and Ig2 in pink. **b** Atomic model of the Fab–Ig2 complex in cartoon representation, highlighting the pincer-like interaction. **c** Surface representation of the Ig2 domain showing the Fab2513 footprint, with contact residues highlighted in dark red. **d** Close-up view of interactions involving CDRH1-S32, N33 and Y35 (blue and black) with IgLON5 (orange and pink). **e** Close-up view of interactions involving CDRH2-Y52 and F53 (blue and black) on IgLON5 (orange and pink). **f** Close-up view of interactions involving CDRH3-M99 and F100 (blue and black) with IgLON5 (orange and pink). **g** Heatmap showing interactions between the heavy chain of Fab2513 and residues of the Ig2 domain involved in binding. Residues are numbered according to Kabat. **h** Two-dimensional representation of the heavy chain sequence of Fab2513 and part of the Ig2 domain of IgLON5. Dashed lines indicate hydrogen bonds and salt bridge interactions. The amino acids involved in the interaction are in bold. **i** Fab–IgLON5 mutants interaction assessed by BLI. The Fab was immobilized on FAB2G biosensor and exposed to IgLON5 mutants at 120 nM. Binding responses (nm shift) were recorded for wild-type protein (in red) or IgLON5 alanine mutants with _165_TWR_167_ in violet _172_GFT_174_ in green, _180_LEI_182_ in blue, and _196_VTH_198_ in orange. The sensorgram shown is representative example of two independent experiments (*n* = 2). **j** UCA–IgLON5 interaction assessed by biolayer interferometry (BLI). The Fab UCA was immobilized on FAB2G biosensor and exposed to IgLON5 at increasing concentrations (15–120 nM). Binding responses (nm shift) were recorded.

To investigate the epitope and paratope involved in the interaction, we performed local refinement of the EM map using a mask encompassing Fab2513 and the second Ig domain of IgLON5. The resulting map reached 3.5 Å resolution, allowing clear identification of the residues involved in the interaction (Fig. 4a, Table 1, Supplementary Figs. 6 and 7). The Fab adopts a pincer-like configuration to engage the second Ig domain of IgLON5, with the heavy and light chains clasping the domain from opposite sides (Fig. 4b). This interaction is mediated by a compact and highly complementary interface spanning residues 163–205 of IgLON5, burying a total surface area of 680 Å² (Fig. 4c). All three CDRs of the heavy chain and the third of the light chain are involved in binding. The VH provides the dominant contribution to binding with 620 Å^2^ and establishes an extended network of polar and hydrophobic interactions across the Ig2 domain of IgLON5. The first CDR is strongly involved in the interaction accounting for half of the buried surface area, with four residues making specific bonds. The CDRH1-S32 (germline encoded) and CDRH1-N33 make hydrogen bounds with T163/T174, and T165/S175, respectively. The CDRH1-Y35, also encoded in the germline, interacts with L169, D171, F173, T174 and Q194 (Fig. 4d). Within the CDRH2, residues Y52 (germline encoded) and F53 interact with V196 and H198, while Y58 (germline encoded) forms a contact with R167 (Fig. 4e). The CDRH3 forms a central interaction network involving CDRH3-N97, which forms non-conventional H-bonds (CH–O/N), interactions with G172, F173 and additional contacts with T174. The CDRH3-R98 and M99 further stabilize this region through CH–O/N and van der Waals interactions with F173 and T174, while F173 is also engaged in apolar contacts with CDRH3-M99 (Fig. 4f). Surrounding this polar core, extensive hydrophobic and proximal contacts reinforce the interface, particularly around the G172, F173 and T174 segment (Fig. 4g). The aromatic and aliphatic residues from CDRH1-Y35, CDRH2-Y52, F53, and CDRH3-M99, F00 pack against W166, R167, Q168, L169, D171, L180, Q181, I182, Q189, Y193, V196, H198, and P205, creating a tightly interlocked and shape-complementary surface (Fig. 4h). The light chain plays a supportive but significant role by reinforcing the same epitope hotspot. CDRL3-Y91 forms a hydrogen bond with D171 and establishes proximal contacts with G172, while CDRL3-T93 contributes additional interactions with D171, complementing the heavy-chain hydrogen-bond network and stabilizing the central binding region. Despite the high structural homology within the IgLON family, mAb2513 does not exhibit cross-reactivity with other members. To explore the molecular basis of this specificity, we aligned the amino acid sequences of the different IgLON proteins. We observed that antibody binding is centered on the highly variable (β12–β12′) loop, which represents the most divergent region among IgLON family members (Supplementary Fig. 9).

To validate the contact residues identified by cryo-EM analysis, we generated a series of IgLON5 mutants. Four clusters of three amino acids involved in the interaction were individually mutated to alanine: _165_TWR_167_, _172_GFT_174_, _180_LEI_182_, and _196_VTH_198._ We then evaluated Fab2513 binding by BLI. As expected, mutation of these contact residues abolished Fab2513 binding (_165_TWR_167_, _172_GFT_174_, and _180_LEI_182_) or strongly reduced it (_196_VTH_198_) (Fig. 4i and Supplementary Fig. 10).

Our structural data also indicate that the interaction is predominantly mediated by germline-encoded residues, as seven of ten unmutated residues in CDRH1 and CDRH2 directly contribute to the binding interface. To explore the contribution of somatic mutations to IgLON5 recognition, we inferred the unmutated common ancestor (UCA) of mAb2513. The UCA possesses an identical CDR3 but differs from the mature antibody by 16 and 8 amino acid changes in the heavy and light chains, respectively (Fig. 1f). We next evaluated the UCA affinity using BLI. We detected an interaction between UCA_2513_ as Fab and IgLON5, with a calculated K_D_ value of 2.8 ± 0.03 × 10^−9^ M (Fig. 4f). This result demonstrated that the UCA retained detectable binding to IgLON5, although with reduced affinity compared to the affinity matured Fab. The reduced affinity of the UCA was mainly attributable to a higher dissociation rate, *k*_off_: 5 × 10^−4^ s^−1^ compared with *k*_off_: 2 × 10^−7^ s^−1^ for the mature mAb2513. This indicates that somatic mutations are not strictly required for IgLON5 recognition but instead contribute to stabilizing the antibody–antigen complex. This finding suggests that IgLON5 recognition is largely driven by germline-encoded features, raising the possibility that autoreactive antibodies can emerge from the naïve repertoire with limited affinity maturation. Our results further indicate that Fab binding does not sterically hinder IgLON5 homo-interactions or heterodimer formation but instead may stabilize pre-existing dimers and promote higher-order multimerization through cross-linking of adjacent IgLON5 molecules. Thus, the structural organization of IgLON5 and the spatial orientation of its extracellular domains support a model in which antibody binding is fully compatible with, and may even facilitate, IgLON5 clustering at the plasma membrane, consistent with the IgLON5 and IgLON4 crosslinking observed in the presence of the mAb.

## Discussion

An increasing number of central nervous system disorders are now recognized as antibody-mediated diseases characterized by autoantibodies directed against extracellular domains of neuronal or glial surface antigens^39,40^. These autoantibodies can directly interfere with protein function, alter synaptic signaling, or induce receptor internalization, leading to neuronal dysfunction^22,40^. Unlike classical paraneoplastic syndromes targeting intracellular antigens, these conditions are frequently responsive to immunotherapy, highlighting their primarily antibody-mediated pathogenic mechanisms. Among them, anti-IgLON5 disease is unique in that it lies at the interface between autoimmunity and neurodegeneration and therefore entails an additional layer of complexity^18^. Understanding the provenance, repertoire and serological consequences of anti-IgLON5 B cells is essential for delineating the pathogenesis of the disease and for therapeutic insights. However, most previous studies have relied on polyclonal IgG purified from patient sera, limiting the ability to dissect antigen specificity at the monoclonal level.

Here, we report, the first sequence and high-resolution structure of a human monoclonal antibody binding IgLON5 with high affinity, providing direct insight into the molecular basis of antigen recognition in this disease. Our analysis indicates that the patient’s B cell repertoire is highly diverse, with no evidence of dominant clonal expansion. This response may arise through bystander T cell activation, engagement of Toll-like receptors in a proinflammatory B cell environment or through widespread activation of diverse B cell clones by foreign or self-antigen^41^. The identification of a modest, but statistically significant increase in somatic hypermutation frequency compared to healthy donors might suggest that high affinity anti-IgLON5 antibodies derive from a sustained antigen stimulation, a finding also in agreement with the predominance of IgG4 observed^23^. Among the identified clones, one IgG4 antibody using the IGHV4-39 germline gene was selected for structural and functional characterization. The IGHV4-39 gene has been recurrently associated with autoreactive antibodies in systemic autoimmune diseases such as systemic lupus erythematosus, antiphospholipid syndrome, and multiple sclerosis^42–44^. Thus, suggesting that intrinsic features of this germline gene may predispose certain B cell clones toward self-reactivity. However, the absence of marked clonal expansion indicates that autoreactivity in anti-IgLON5 disease is unlikely to arise from a single dominant lineage and may instead contribute to the clinical heterogeneity observed across patients.

The structural analysis reveals that the antibody recognizes a conformational epitope located within the second Ig-like domain of IgLON5. This mode of binding highlights the importance of native protein folding in antibody recognition and is consistent with the absence of reactivity against other members of the IgLON family despite their high degree of structural homology^8^. Sequence comparison indicates that the antibody footprint overlaps with the β12–β12′ loop of the Ig2 domain, one of the most divergent regions among IgLON proteins, supporting the idea that subtle structural variations within surface-exposed loops drive antigen specificity. These findings provide a molecular explanation for the selective targeting of IgLON5 and reinforce the notion that autoimmune responses against neuronal surface proteins can achieve high specificity despite conserved protein architectures.

The Fab engages the Ig2 domain of each protomer in an antiparallel manner within the IgLON5 dimer, recapitulating observations previously reported with purified patient IgG^27^. Notably, our data demonstrate that Fab binding does not disrupt the IgLON5 dimerization interface but instead is compatible with, and may promote, higher-order homo- or hetero-multimerization. Such antibody-induced clustering has been reported to trigger receptor internalization and may contribute to neuronal dysfunction and tau pathology^30^. In this context, an important question raised by our structural findings is the potential stoichiometry of full-length IgG4 binding to dimeric, membrane-associated IgLON5. Several configurations can be envisioned. One IgG molecule could bridge two IgLON5 protomers within a dimer or, perhaps more plausibly given the pre-existing dimeric arrangement and membrane constraints, engage IgLON5 molecules from neighboring dimers, thereby promoting higher-order clustering. Alternatively, each protomer within an IgLON5 dimer could be bound by a distinct IgG molecule, resulting in a 2:2 IgG arrangement that may further stabilize or organize multimeric assemblies. These possibilities are particularly relevant for IgG4 antibodies, whose functional monovalency after Fab-arm exchange may reduce, but not necessarily abolish, their capacity to cross-link membrane IgLON5. This is consistent with our observation that full-length mAb2513 in the IgG4 format enhances IgLON5 association less efficiently than the corresponding IgG1. Similar principles have recently been described for anti-NMDAR autoantibodies, where epitope location and antibody–receptor stoichiometry influence functional outcomes, including receptor modulation and internalization^45^. Future studies using full-length antibodies, membrane-tethered IgLON5, and quantitative imaging or structural approaches will be required to define the precise stoichiometry of IgG4–IgLON5 complexes and determine how this geometry contributes to IgLON5 clustering and downstream pathogenic effects.

The paratope of mAb2513 involves all three CDRs of the heavy chain and CDR3 of the light chain, with several contacts mediated by residues located in CDRH1 and CDRH2. Because these regions are partly germline encoded, this binding mode suggested that the naïve precursor of mAb2513 might already possess intrinsic reactivity toward IgLON5. Consistent with this hypothesis, the inferred unmutated common ancestor of mAb2513, in which the mature CDR3 sequences are preserved while somatic mutations in the V and J genes are reverted to their germline configuration, retained detectable binding to IgLON5. This indicates that initial antigen recognition is supported by a combination of germline-encoded residues and the conserved CDR3 loops. However, the reduced affinity of the UCA compared with the mature antibody, mainly due to a faster dissociation rate, suggests that somatic mutations acquired during affinity maturation stabilize the interaction with IgLON5. These findings support a model in which low-affinity self-reactivity may be present at the precursor stage and subsequently strengthened through affinity maturation. This mechanism is reminiscent of recent observations in anti-NMDAR autoimmunity, where germline or near-germline BCRs with low-affinity self-reactivity have been implicated in the emergence of pathogenic antibodies in the context of human breast cancer^46^ and have also been described in several autoimmune encephalitides^47^.

Our study has some limitations, the structural and functional analyses are based on a mAb derived from a single patient, and it remains to be determined whether similar epitopes and binding modes are shared across individuals. In addition, while our data provide a structural framework for understanding antibody binding, the downstream cellular consequences of this interaction require further investigation. Future studies should aim to characterize additional mAbs from multiple patients to assess the degree of convergence in epitope targeting and germline usage. Finally, integrating structural insights with in vivo models may help clarify how antibody-mediated perturbations of IgLON5 translate into the complex clinical phenotype of the disease.

In conclusion, our work provides a high-resolution view of a human monoclonal antibody targeting IgLON5 and reveals a mechanism of antigen recognition that preserves IgLON5 dimerization while likely altering its organization at the cell surface. This reorganization may contribute to downstream pathogenic effects, such as tau phosphorylation, missorting, and over time tau deposition and neurodegeneration. These findings redefine current models of pathogenicity in anti-IgLON5 disease and support a paradigm in which antibody-mediated functional modulation, rather than direct structural disruption, drives neuronal dysfunction in an autoimmune disorder of the central nervous system.

## Methods

### Patient and sample description

PBMCs were obtained from a 60-year-old male diagnosed with anti-IgLON5 disease. The patient presented a 7-year history of sleep disturbances characterized by abnormal nocturnal movements and behaviors, mild sleep-onset and maintenance insomnia, and severe daytime somnolence with episodes of unintended sleep. During sleep, he exhibited loud snoring, frequent vocalizations, groaning, and complex motor behaviors, including punching, kicking, jerking, and gesturing. Additional neurological symptoms included mild dysphagia with occasional choking episodes, gait unsteadiness with infrequent falls, and persistent daytime motor restlessness. Neurological examination revealed distal choreic movements affecting both the upper and lower limbs, as well as abnormal postural reflexes on the pull test. Overnight video polysomnography demonstrated NREM and REM parasomnia, accompanied by stridor and severe obstructive sleep apnea, with an apnea–hypopnea index of 44 events per hour. Laryngoscopic evaluation revealed mild bilateral vocal cord paresis. Routine blood tests, cerebrospinal fluid analysis, and brain MRI were unremarkable. IgLON5 antibodies, however, were detected in both serum and cerebrospinal fluid, confirming the diagnosis of anti-IgLON5 disease. HLA typing was positive for the DRB1*10:01 and DQB1*05:01 haplotype. The patient received immunotherapy with high-dose intravenous corticosteroids followed by rituximab, without clinical improvement. Continuous positive airway pressure therapy effectively eliminated stridor and obstructive sleep apnea and improved daytime sleepiness, while abnormal sleep-related movements and behaviors persisted. The patient repertoire comprised 3.1% IgG1, 4.1% IgG2, 1.1% IgG3, and 0.5% IgG4 of IgLON5 specific clones. Written informed consent for the storage and research use of PBMC sample was obtained from the patient. PBMC collection was approved by the Ethics Committee of Hospital Clínic de Barcelona, Spain (2018-0336 ER-01).

### Cell Lines

Expi293F cells (ThermoFisher, Cat No# A14527), a human embryonic kidney cell line adapted for suspension growth, were obtained from Thermo Fisher Scientific and cultured in Expi293 Expression Medium (Gibco, Cat No #A1435101) and HyCell TransFx-H liquid medium (Cytiva, Cat No. #SH30939.02) supplemented with GlutaMax (Gibco, Cat No #10860311), Sodium pyruvate (ThermoFisher, Cat No# 11360070), and Poloxamer 188 (Gibco, Cat No #24040032). SH-SY5Y cells, a human neuroblastoma cell line, were obtained from ATCC (CRL-2266) and cultured in a 1:1 mixture of ATCC-formulated Eagle’s Minimum Essential Medium (ATCC, 30-2003) and F-12 Medium (Gibco, Thermo Fisher Scientific, Cat No#11765054), supplemented with 10% FBS (Sigma-Aldrich, Cat No#A5256701).

### Staining of hiPSC-derived neurons

Neurons were obtained through differentiation of human induced pluripotent stem cell (hiPSC)-derived neural precursor cells (NPCs) and characterized, as previously described (cell line ID: LNISi002 -B)^35,48^. To assess both extracellular and intracellular mAb binding to hiPSC-derived neurons, cells were fixed with 3% paraformaldehyde (PFA, Electron Microscopy Sciences, Cat No#15710), 0.1% glutaraldehyde (Thermo Fisher Scientific, Cat No#A17876.AE), and 2% sucrose (Thermo Fisher Scientific, Cat No# J65148.36) for 10 min at room temperature (RT), followed by staining as previously described^49^. Briefly, after permeabilization and blocking of non-specific binding sites using a permeabilization/blocking buffer (Perm./Block buffer: 5% normal goat serum (Jackson ImmunoResearch, Cat No#005-000- 121), 0.2% saponin (Sigma-Aldrich, Cat No#47036), and 0.1% Triton X-100 (Sigma-Aldrich, Cat No#T9284) in PBS) cells were incubated overnight at 4 °C with either anti-MAP2 at 4 µg/mL (Abcam, Cat No#ab5392), anti-IgLON-5 (mAb2513, this manuscript) at5 µg/mL or Palivizumab (isotype control; 5 µg/mL), all antibodies diluted in Perm./Block buffer. Subsequently, biotinylated human anti-IgG secondary antibodies at 1:100 (Invitrogen, Cat No#31774; 1:100) were applied for 30 min at RT to detect bound primary antibodies. Following extensive PBS washes, phycoerythrin (PE)-labeled streptavidin at 1:1000 (BD, Cat No#554061) was added and incubated for 30 min at RT. Cells were then counterstained with 4′,6-diamidino-2-phenylindole (DAPI, Sigma-Aldrich, Cat No#D9542) diluted in PBS for 10 min at RT. All samples and controls were performed in duplicates. Fluorescence microscopy of stained cells was automated using a high-performance EVOS M7000 imaging system. Four images were acquired per well. Post-acquisition image processing was performed using ImageJ software (NIH, v1.52t). Image acquisition and processing were conducted using identical parameters for specific antibodies and corresponding controls.

### Cell sorting

PBMCs were stained on ice for 20 min with the following fluorochrome-labeled mouse monoclonal antibodies: CD3-APC/Cy7 at 1:20 (clone HIT3a, Cat No#300317, BioLegend), CD20-PE-Cy7 at 1:20 (clone 2H7, Cat No#302312, BD Biosciences), and anti-human IgD PE-CF594 at 1:20 (BD Biosciences; Cat No#562540; Clone IA6-2). Cells were sorted to over 98% purity on a FACS Aria III (BD) using the following gating strategy: circulating memory B cells were sorted as CD3–, CD20+ IgD-, IgLON5 labeled with Alexa647 + /-. The FACS-sorted B cells were collected in 6 μl FCS in Eppendorf tubes that were pre-coated overnight with BSA (Sigma-Aldrich).

### IgLON cell-surface association assay

Biotinylated IgLON5 was preincubated with streptavidin-PE (Thermo Fisher Scientific, Cat No#12-4317-87) for 15 min on ice. The resulting complexes were then incubated for 5–10 min on ice with either an isotype control antibody (palivizumab), mAb2513 IgG1, mAb2513 IgG4, or Fab2513 before being added to the cells. IgLON5 association at the cell surface was assessed by flow cytometry in the presence of LIVE/DEAD Fixable Violet Dead Cell Stain at 1:1000 (Thermo Fisher Scientific, Cat No# L34964). Geometric mean fluorescence intensity (gMFI) was quantified using BD FACSDiva software and normalized to the frequency of the parent population. Data visualization and statistical analyses were performed using GraphPad Prism version 10.4.1.

### Single-cell BCR-seq library preparation and sequencing

Single-cell 5′ V(D)J libraries were generated using Chromium Next GEM Single Cell V(D)J Reagent kit v3 (10X Genomics) according to the manufacturer’s protocol. Paired heavy and light chain BCR libraries were prepared from the sorted B cell populations. Briefly, up to 20,000 memory B cells per well of 10X chip were loaded in the 10X Genomics Chromium Controller to generate single-cell gel beads in emulsion. After reverse transcription, gel beads in emulsion were disrupted. Barcoded complementary DNA was isolated and used for the preparation of BCR libraries. All steps were performed according to the manufacturer’s instructions. The purified libraries from four biological replicates of donor D1 (one IgLON5 positive and three negative) were pooled separately and sequenced on the Element AVITI System as per the instructions provided in 10X Genomics user guide for the read length and depth.

### Paired BCR repertoire

V(D)J annotation was performed using cellranger vdj (v10.0) on each sample individually, after which sequences and annotations were pooled into a single filtered_contig.fasta and filtered_contig_annotations.csv file for downstream processing within the Immcantation framework (http://immcantation.org; Change-O v1.3.0)^50^. Sequences were subsequently annotated using IgBLAST v1.22^51^ against the IMGT database^52^ (accessed 8 December 2025). Only productive rearrangements were retained, and heavy and light chain sequences were separated into independent files prior to clonal analysis^53^. Clone assignment was performed using the Change-O DefineClones function, grouping sequences by IGHV gene, IGHJ gene, and junction sequence similarity (distance threshold = 0.15). Clone definitions were subsequently verified and corrected using the light_cluster function, which leverages light chain pairing information associated with each heavy chain clone. Germline sequences were reconstructed using the CreateGermlines function, and sequences harboring incomplete V(D)J rearrangements or in-frame stop codons were excluded from further analysis. For downstream repertoire analysis, only heavy chains paired with exactly one κ or one λ light chain were retained. The filtered heavy and light chain sequences were concatenated into a single AIRR format file for use as input for scRepertoire v2.6.2^54^. The same pipeline was applied to two publicly available healthy donor datasets^32^. The three repertoires yielded 9577 paired BCRs for D1 (IgLON5 patient), 7263 for H1, and 7539 for H2. Clonal diversity was assessed using coverage-based rarefaction and extrapolation analysis, implemented via the clonalRarefaction() function from scRepertoire. Sample completeness curves were first generated to evaluate the relationship between the number of sequenced cells and sample coverage for each donor (plot.type = 2). Coverage-based rarefaction curves were then computed for two Hill diversity orders: species richness (Hill q = 0), which accounts for clonal richness alone, and Shannon diversity (Hill q = 1), which incorporates both clonal richness and evenness (plot.type = 3). For all metrics, rarefaction (interpolation) was performed up to the observed sample size, and extrapolation was extended beyond it. Confidence intervals were estimated using bootstrap resampling (95% CI, n.boots = 50). V gene usage was visualized using the vizGenes() function from scRepertoire. Mutation frequency was calculated as the number of residue differences between the BCR protein sequence and its inferred germline sequence across the FWR1, CDR1, FWR2, CDR2, and FWR3 regions of the VH gene. Clonal network of CDRH3 sequences was constructed using the clonalCluster() function from scRepertoire v2.2.1. Briefly, clonal family identifiers derived from the Immcantation workflow were used as V gene input, replacing the VH gene in the CTgene metadata, for edit-distance-based clustering (parameters: chain = “IGH”, sequence = “aa”, threshold = 0.81). Statistics were conducted using R Statistical Software (v4.4.1). Sequences of the unmutated common ancestor (UCA) of the VH- and VL-coding genes were inferred with Antigen Receptor Probabilistic Parser (ARPP) UA Inference software^55^.

### Recombinant antibody production

Expi293 cells (Thermo Fisher, Cat No# A14527) were diluted to a cell density of 2.5 × 10⁶ cells/mL in cell culture medium. Heavy chain and light chain plasmids were complexed with Polyethyleneimine (Chemie Brunschwig AG, Cat No# POL24765) and added to the cells. On day five, cells were cleared from cell culture media by centrifugation at 10,000 *g* for 30 min and subsequently passed through a 0.22-μm filter. The supernatant containing the recombinant antibody was applied on protein A resin (Cytiva, Cat No#17040301) at 4 °C. The resin was washed with 10 column volumes (CV) of phosphate-buffered saline (PBS) and antibody eluted with 3 CV of 100 mM glycine (Sigma-Aldrich, Cat No#G7126) pH 2.9^56^. The acidic pH of the eluted antibody solution was increased to approximately 7 by the addition of 1 M Tris pH 8.0 (Sigma-Aldrich, Cat No#T1503). The antibody solution was buffer exchanged into PBS with successive rounds of centrifugation, filtered, and stored at −80 °C.

### Fragment antigen binding (Fab) generation

For Fab production, the heavy chain was engineered at the hinge region to include a two–amino acid glycine–serine linker, followed by a C-terminal six-histidine tag and a stop codon. Light and mutated heavy chains were transfected as described in the previous section. Cell supernatant was harvested five- or six-days post transfection and purified by IMAC chromatography, followed by size exclusion chromatography on a Superdex 200 Increase 10/300 GL column (Cytiva, Cat No#28990944).

### Recombinant proteins production

DNA encoding either the full length ORFs or truncated version of IgLON proteins (residues 1–316) fused to human IgG1 Fc domain or a linker followed by tandem Strep tag and 6×His tag were ordered from Twist Bioscience (sequence of construct are listed in Supplementary Fig. 11). The plasmids were transfected into Expi293 cells using polyethyleneimine (Chemie Brunschwig AG, Cat No# POL24765) as described above. The recombinant proteins were purified by either IMAC or Protein A affinity chromatography, followed by digestion with immobilized papain agarose (Thermo Fisher Scientific, Cat No#20341) to remove the Fc fragment. The cleaved IgLON were further purified by size-exclusion chromatography on a 200 Increase 10/300 GL column (Cytiva, Cat No#28990944). Fractions containing the IgLON proteins were pooled, sterile-filtered, snap-frozen, and stored at −80 °C.

### Biolayer interferometry

The biolayer interferometry experiments using IgLON5 were performed as follows. All experiments were performed in reaction buffer (PBS pH 7.4  +  0.01% (w/v) BSA (Sigma-Aldrich, Cat No#A3912) +  0.002% (v/v) Tween 20 (Sigma-Aldrich, Cat No#003005) at room temperature (RT) using an Octet K2 instrument (ForteBio). FAB2G (Fortebio, Cat No#18-5125) biosensor probes were first equilibrated in reaction buffer for 600 s. Fab fragments were diluted to 5 µg/ml in reaction buffer and immobilized onto the FAB2G probes for 600 s, followed by a wash for 300 s in reaction buffer. The binding of IgLON5 to the Fab was then measured at various concentrations for 400 s, followed by dissociation for 500 s in reaction buffer. Analysis was performed using the Octet software and GraphPad Prism version 9.0.

### Cryo-EM sample preparation

IgLON5/Fab complexes were prepared using a stock solution at 2 mg/ml by incubating IgLON5 protein with a tree-fold molar excess of Fab2315. To prevent interaction of the complex with the air-water interface during vitrification, the sample was incubated with 0.005% (w/v) fluorinated Fos-Choline 8 (FC8) (Cat No#FERA65499, Thermo Fisher Scientific). Next, sample was applied to plasma cleaned Quantifoil 1.2/1.3 300 -mesh Cu holey carbon grids (Electron Microscopy Sciences). The grid was blotted in an automatic plunge freezing apparatus Vitrobot MarkIV (Thermo Fisher, Hillsboro, USA) to control humidity and temperature^53^.

### Cryo-EM data collection

Grids were screened for particle presence and ice quality on a TFS Glacios microscope (200 kV), and the best grids were transferred to a TFS Titan Krios G4. Cryo-EM data were collected using a TFS Titan Krios G4 transmission electron microscope, equipped with a Cold-FEG on a Falcon IV detector in electron counting mode. Falcon IV gain references were collected just before data collection. Data were collected using TFS EPU v2.12.1 utilizing the aberration-free image shift protocol, recording 4 micrographs per ice hole. Movies were recorded at a magnification of ×81,000, corresponding to the 0.83 Å pixel size at the specimen level, with defocus values ranging from −1.2 to −2.4 µm. Exposures were obtained with 17.34 e^−^ Å^−2^ s dose, resulting in an exposure time of approximately 2.75 s per movie. In total, 17,169 micrographs in EER format were collected.

### Cryo-EM data processing and structure fitting

Data processing was performed with cryoSPARC V4.4 including motion correction and CTF determination^57^. Initial particle picking was performed using blob picking on 1,000 micrographs. The extracted particles were sorted by 2D classification, and the resulting classes were used to train DeepPicker, which was then applied to the full dataset^58^. Particles extraction (box size 448 pixels^2^) was carried out using cryoSPARC^57^. Next, several rounds of reference-free 2D classification were performed to remove artifacts and selected particles were used for ab-initio reconstruction and hetero-refinement. After hetero-refinement, 702’836 particles contributed to an initial 3D reconstruction of 3.7 Å resolution (FSC 0.143) with C1 symmetry. Local refinement was performed with mask generated with UCSF Chimera around IgLON5 domain 2 and Fab and generated a map at 3.5 Å resolution. The model of a IgLON5 dimer (PDB ID: 6DLE)^12^ and model of Fab 2315 (obtained withAlphaFold2^59^) were fitted into the cryo-EM maps with UCSF Chimera. These docked models were extended and rebuilt manually with refinement, using Coot and Phenix^60,61^. Figures were prepared in UCSF Chimera, UCSF ChimeraX and Pymol^62^. The numbering of Fab 2315 is based on Kabat numbering of immunoglobulin models^63^. Buried surface area measurements were calculated within ChimeraX and PISA^64^.

### Statistical analysis

Statistics were conducted using R Statistical Software (v4.4.2) and with Prism Graph Pad (v10) using one-way ANOVA with Tukey’s multiple comparisons test.

### Reporting summary

Further information on research design is available in the Nature Portfolio Reporting Summary linked to this article.

## Supplementary information

Supplementary Information

Transparent Peer Review file

Reporting Summary

## Source data

Source Data

## Data Availability

The raw sequencing data files for single-cell VDJ sequencing are available at the European Nucleotide Archive under accession number PRJEB109754. Processed data files (BCR repertoires) are available at Zenodo [https://doi.org/10.5281/zenodo.18925481]. The cryo-EM map and PDB models are available on EMDB with accession numbers EMD-57080 and EMD-57198 and on PDB with the PDB-ID 29CP and 29IN. Source data for Figs. 2 and 4 and for Supplementary Figs. 4 and 10 are provided with the paper. Source data are provided with this paper.

## Code availability

The scripts used in the present study are available on https://github.com/MathildeFogPerez/manuscript-iglon5-D1.
