# Supplementary material for: Structural basis of IgLON5 autoantibody recognition in autoimmune encephalitis
Source: Nat Commun. 2026 Sep 11;17:10734. doi: 10.1038/s41467-026-77812-6 (PMC13658817; doi:10.1038/s41467-026-77812-6)
Supplement: Supplementary file 1 [file 41467_2026_77812_MOESM1_ESM.pdf]

Supplementary Materials for

Structural basis of IgLON5 autoantibody recognition in autoimmune encephalitis

Angelique Roux, Rachel Schelling, David Vinyals Sales, Lidia Sabater, Rahel R. Winiger, Ilayda Senyuz, Alison Lin, Amandine Mathias, Renaud Du Pasquier, Carles Gaig, Josep Dalmau, Mathilde Foglierini and Laurent Perez[#]

#Corresponding author: Laurent Perez

email: laurent.perez@chuv.ch

The PDF file includes Figs. S1 to S11

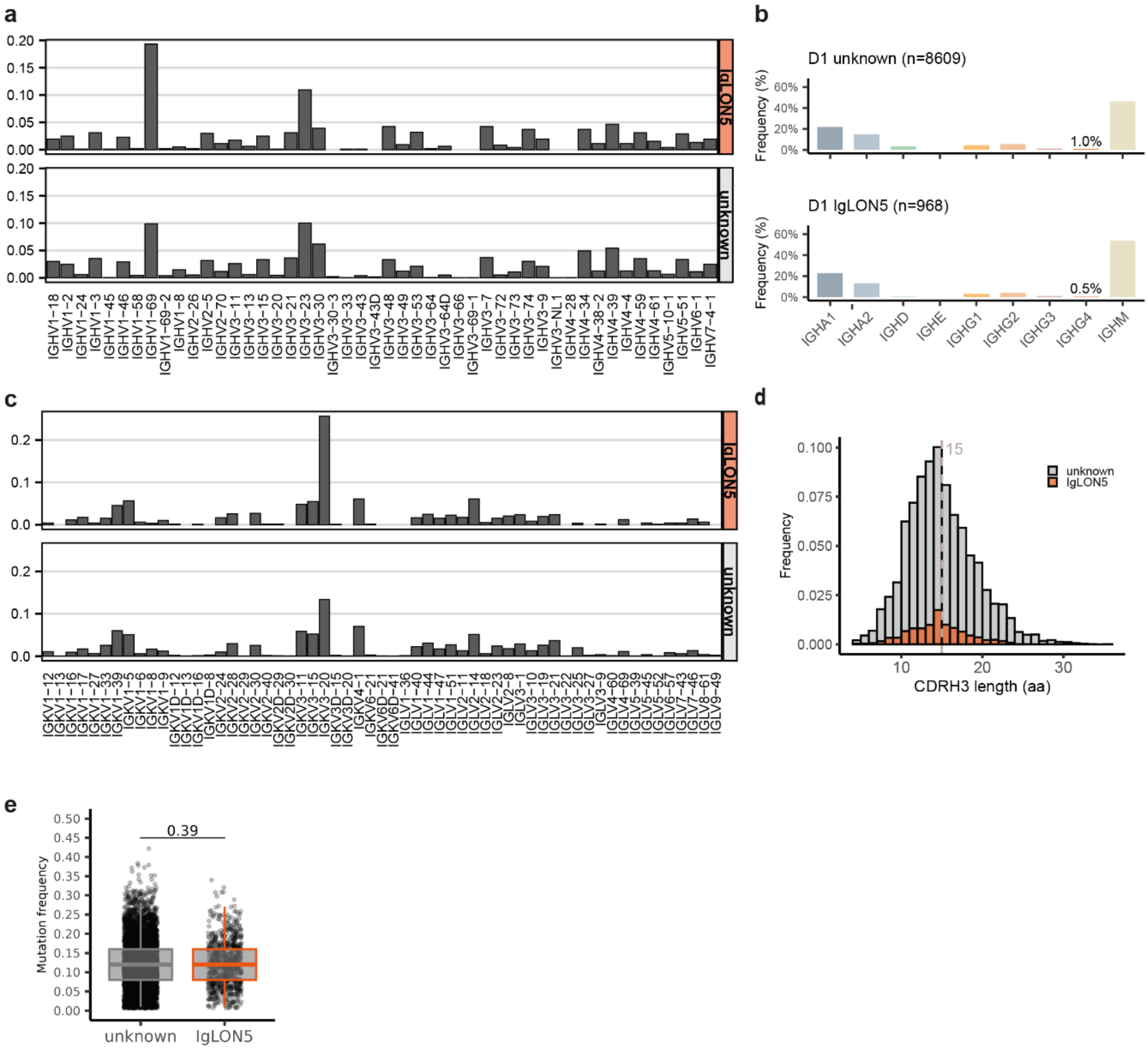

Supplementary Figure 1. Comparison of IgLON5-specific and non-specific B cells from donor D1. (a) Bar chart showing the frequency of VH gene usage among IgLON5-specific BCRs and BCRs of unknown specificity. **(b)** Isotype distribution of IgLON5- specific and non-specific BCRs. The total number of clonotypes per group is indicated in parentheses, and the frequency of IgG4 is annotated on the corresponding bars. **(c)** Bar chart showing the frequency of VK/VL gene usage among IgLON5-specific BCRs and BCRs of unknown specificity. **(d)** Histogram showing the distribution of CDRH3 length in IgLON5-specific and non-specific BCRs. Dashed lines indicate median values. **(e)** Boxplot showing somatic hypermutation frequency (amino acid substitutions relative to the V gene germline) in the two groups. Boxes represent the interquartile range (IQR; 25th–75th percentile), with the central line indicating the median. Whiskers extend to 1.5× the IQR, and individual points beyond this range are shown as outliers. Statistical significance was assessed using a Wilcoxon test (P = 0.39).

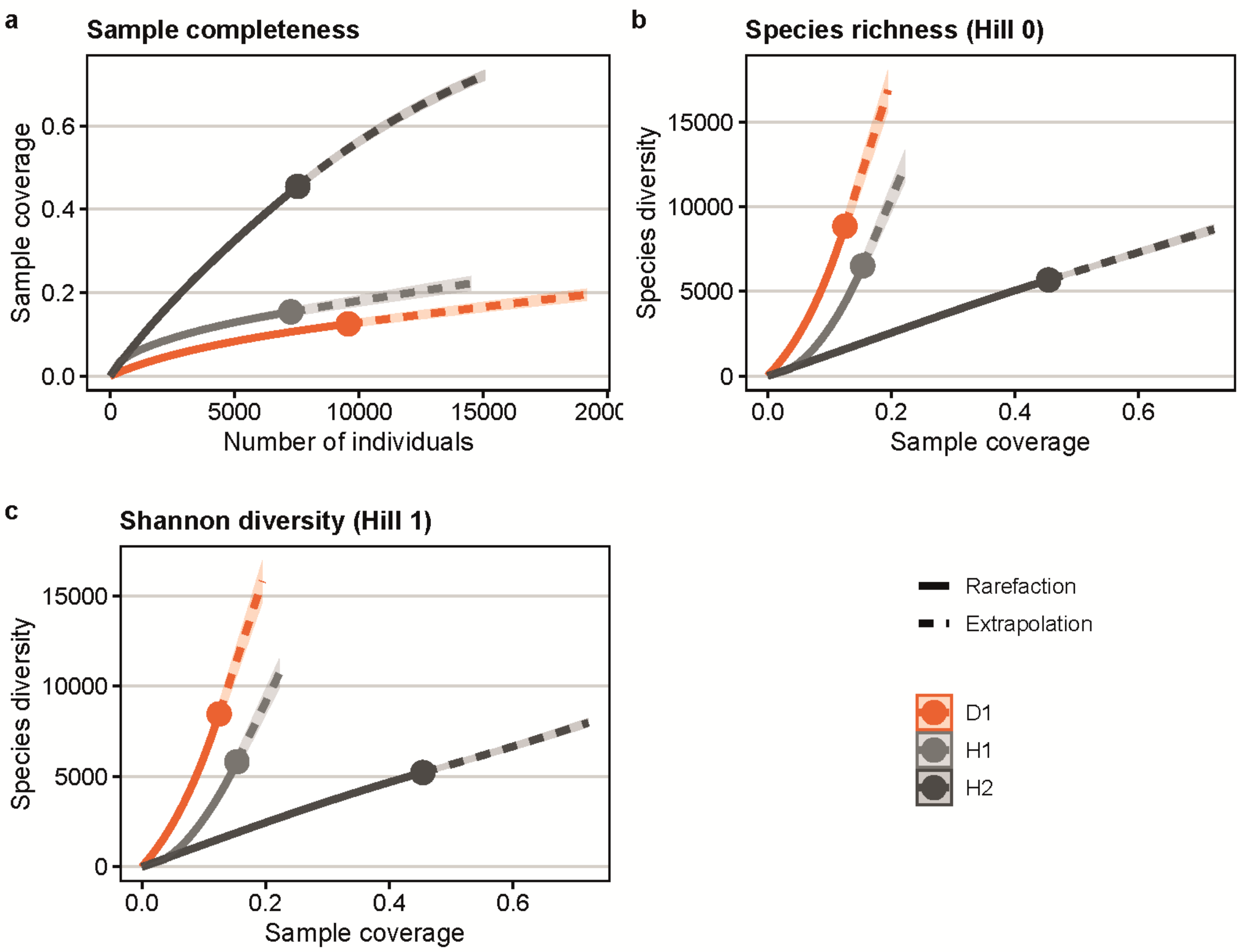

Supplementary Figure 2. BCR repertoire diversity across donors assessed by rarefaction analysis. (a) Sample completeness curves showing the relationship between number of sequenced cells and sample coverage for each donor. The dot indicates the observed sample size. **(b)** Coverage-based rarefaction curves for species richness (Hill q=0), showing estimated clonal diversity as a function of sample coverage. **(c)** Coverage-based rarefaction curves for Shannon diversity (Hill q=1), reflecting both clonal richness and evenness. In all panels, solid lines indicate rarefaction (interpolation) and dashed lines indicate extrapolation beyond the observed sample size. Shaded areas represent 95% bootstrap confidence intervals (n=50 replicates). D1, orange; H1, light grey; H2, dark grey.

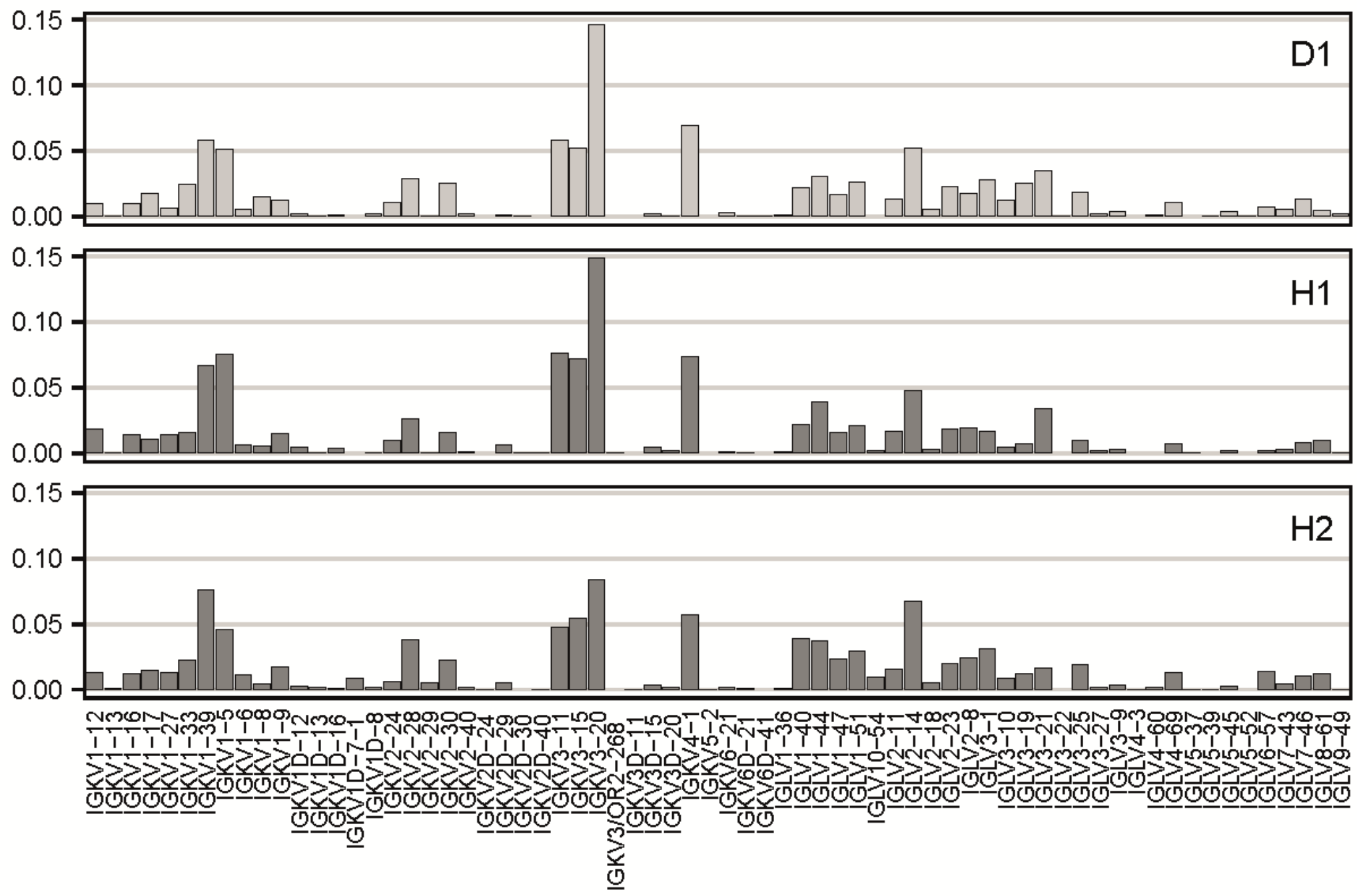

Supplementary Figure 3. Light chain V usage. Bar chart showing the frequency of VK/VL gene usage among paired BCRs in memory B cell populations from anti-IgLON5 patient D1 and two healthy donors (H1 and H2).

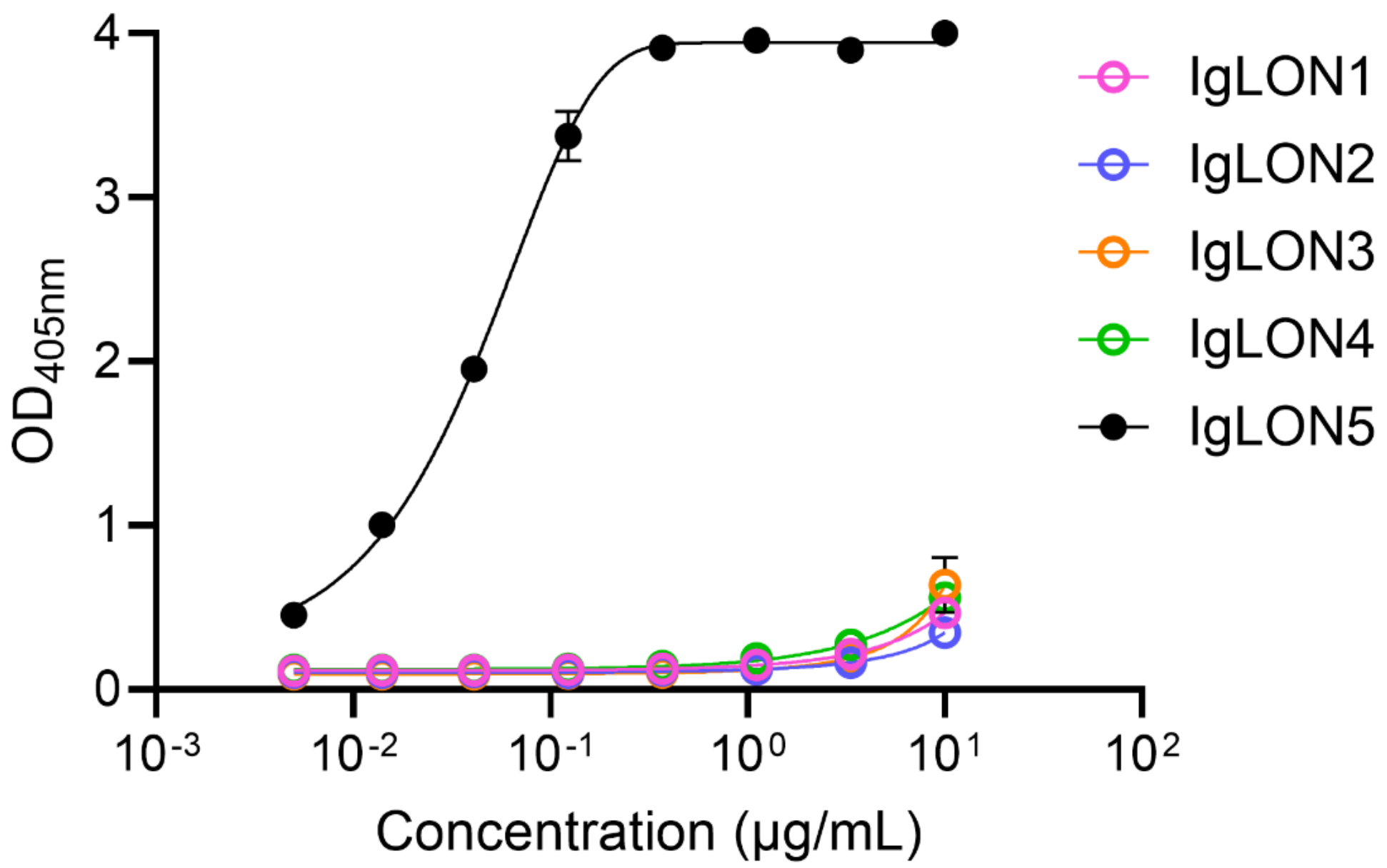

Supplementary Figure 4. Reactivity of mAb2513 with members of the IgLON protein family. Purified IgLON1 (pink circles), IgLON2 (blue), IgLON3 (orange), IgLON4 (green), and IgLON5 (closed circles) were coated onto MaxiSorp plates at 5 µg/mL. Binding of mAb2513 was assessed by ELISA using serial dilutions of the antibody. Data shown are representative of two independent experiments (n = 2).

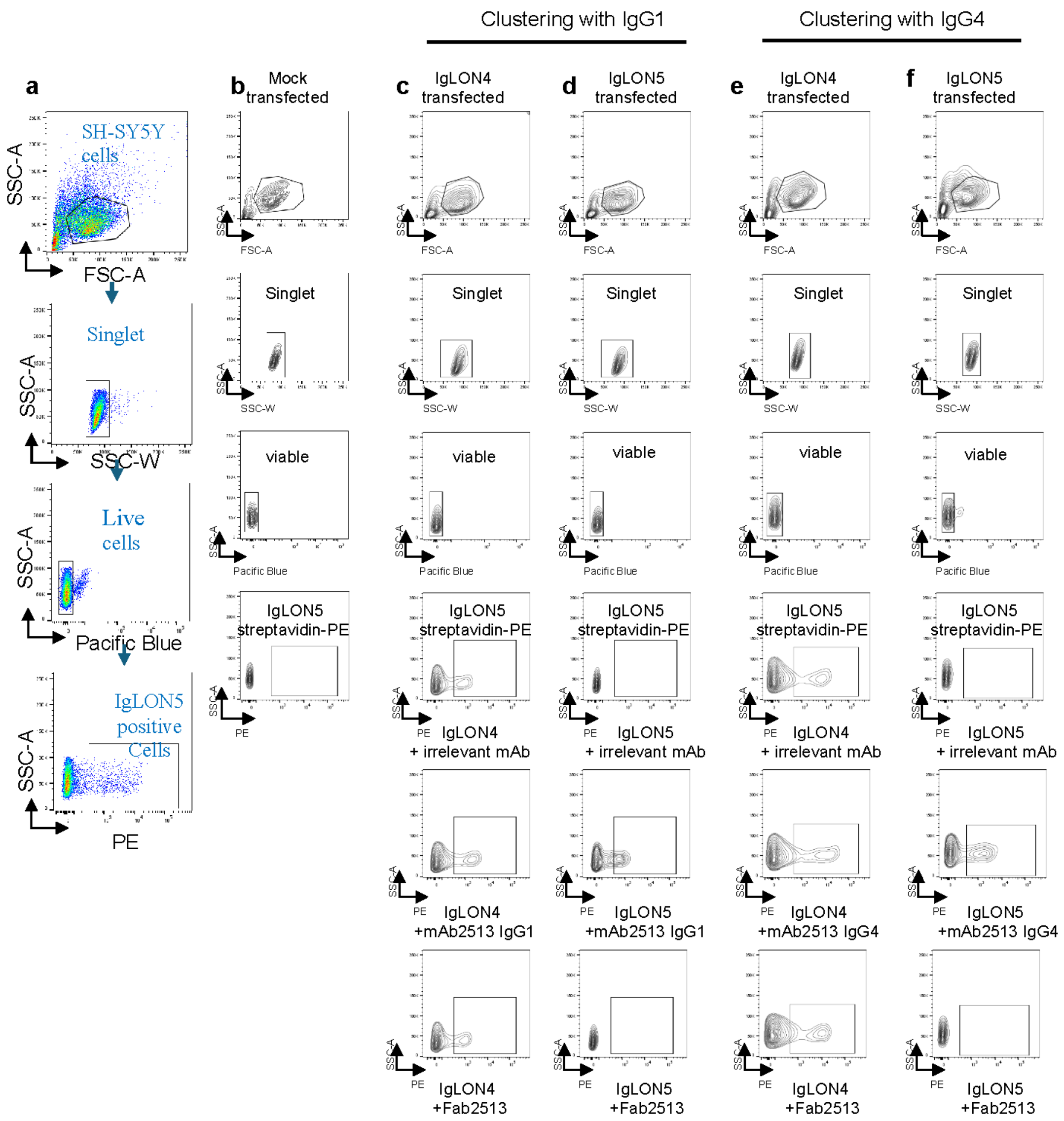

Supplementary Figure 5. Gatting strategy for IgLON5 clustering on SH-SY5Y. (a) Gating strategy to assess IgLON5 cell surface association by flow cytometry. **(b-f)** Representative FACS profiles used to measure geometric mean fluorescence intensity in Figure 2e and Figure 2f. **(b)** SH-SY5Y neuroblastoma cells, transfected with a mock construct, were probed with biotinylated IgLON5 and streptavidin-PE. **(c)** SH-SY5Y neuroblastoma cells, transfected with IgLON4 construct, were probed with biotinylated IgLON5 and streptavidin-PE in presence of an isotype control mAb (palivizumab), mAb2513 as IgG1 or Fab2513. **(d)** SH-SY5Y neuroblastoma cells, transfected with IgLON5 construct, were probed with biotinylated IgLON5 and streptavidin-PE in presence of an isotype control mAb (palivizumab), mAb2513 as IgG1 or Fab2513. **(e)** SH-SY5Y neuroblastoma cells, transfected with IgLON4 construct, were probed with biotinylated IgLON5 and streptavidin-PE in presence of an isotype control mAb (palivizumab), mAb2513 as IgG4 or Fab2513. **(f)** SH-SY5Y neuroblastoma cells, transfected with IgLON5 construct, were probed with biotinylated IgLON5 and streptavidin-PE in presence of an isotype control mAb (palivizumab), mAb2513 as IgG4 or Fab2513Experiments were repeated with three biological replicates (n=3).

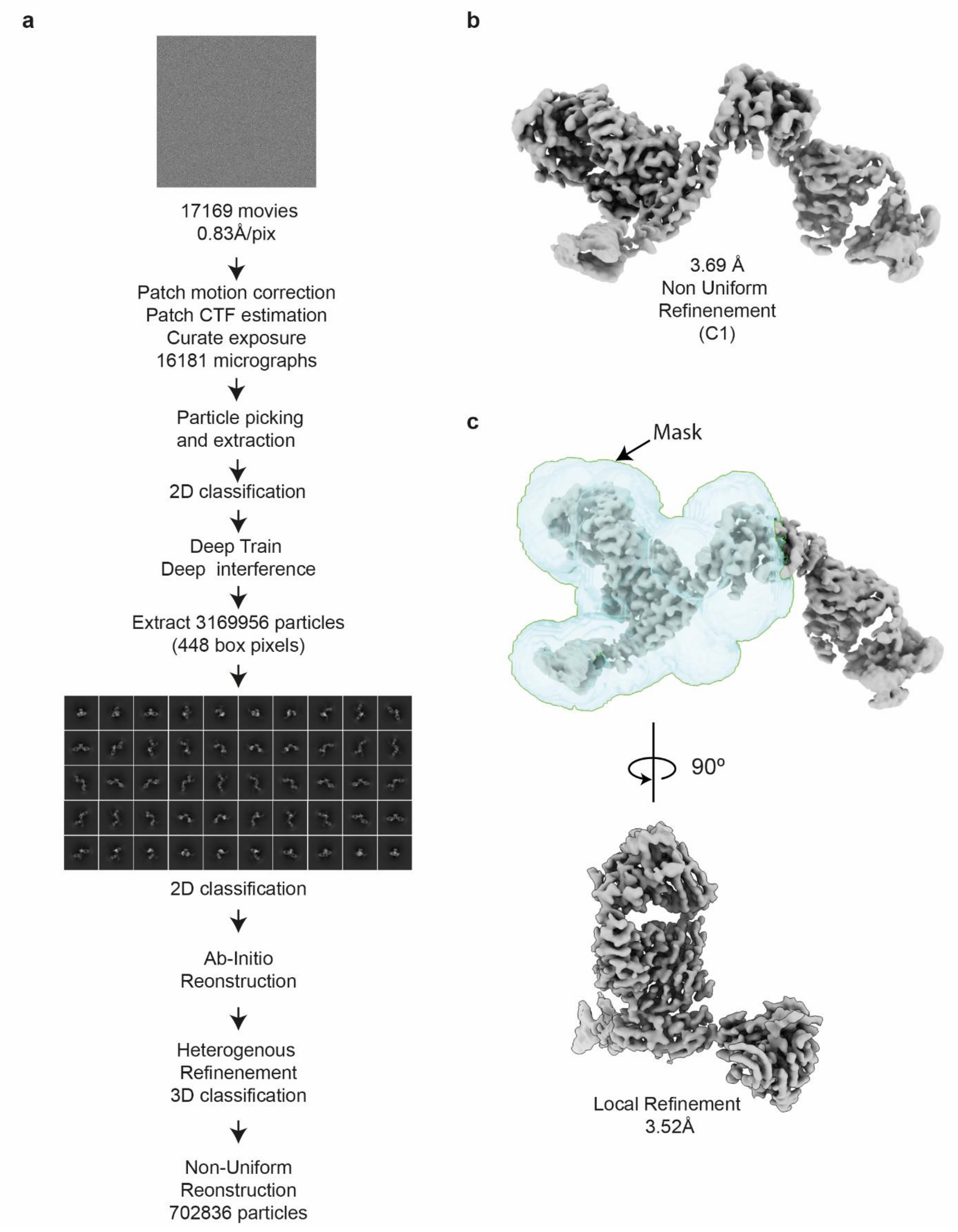

Supplementary Figure 6. Cryo-EM data processing of IgLON5–Fab2513 complex structures. (a) Workflow of single-particle analysis and reconstruction of the IgLON5–Fab2513 complex. Particles displaying clear structural features were selected based on 2D class averages. Multiple rounds of 3D classification were performed without symmetry (C1), and classes with similar conformations were combined for subsequent 3D refinement under C1 symmetry. **(b–c)** Cryo-EM density maps of the complex shown in grey, including the full reconstruction **(b)** and the locally refined map **(c).**

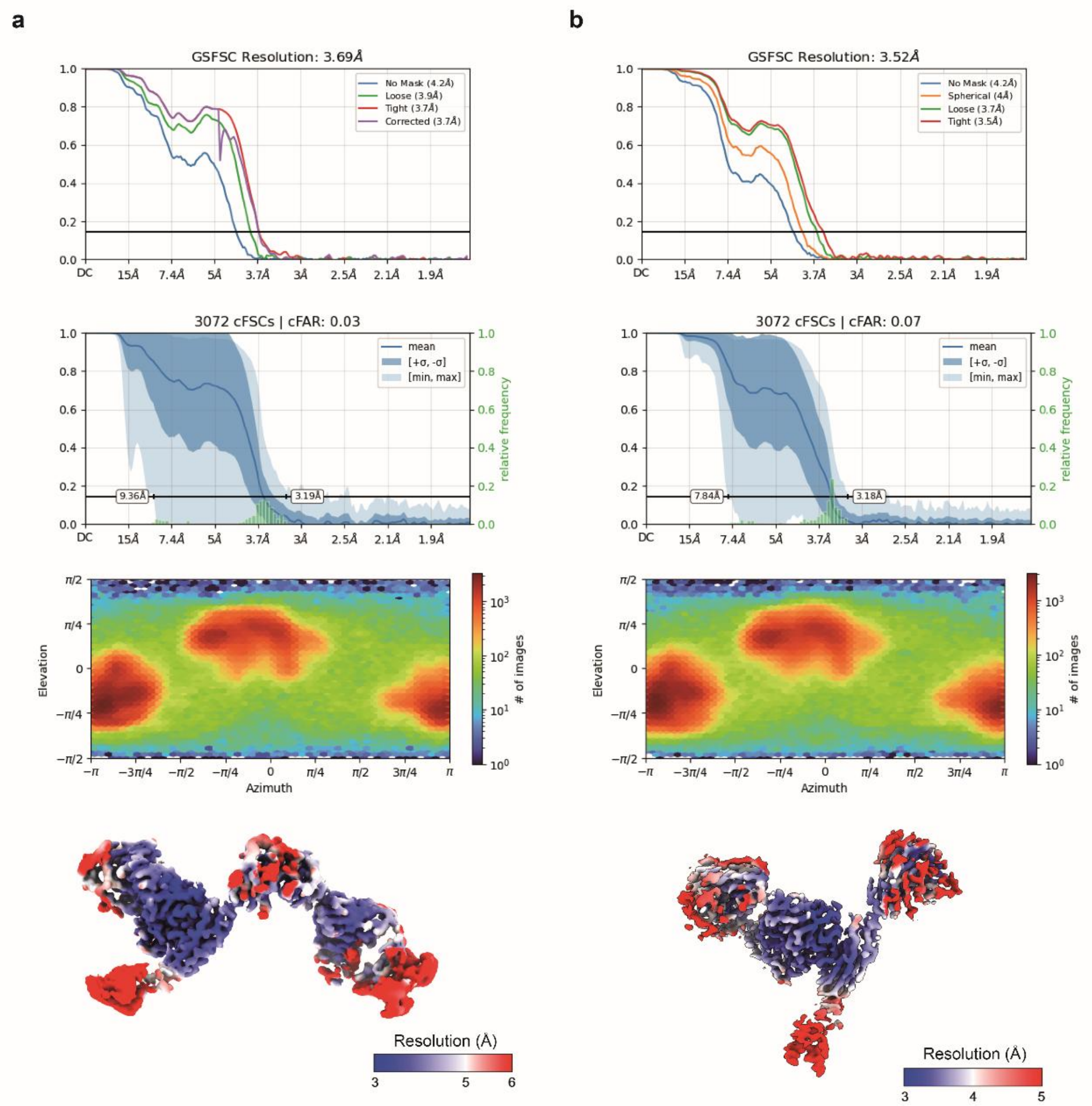

Supplementary Figure 7. Cryo-EM processing and resolution assessment. (a–b) Fourier shell correlation (FSC) curves and Euler angle distributions of particle images for the global and masked cryo-EM maps, together with local resolution maps of the Fab2513–IgLON5 complex for the full reconstruction **(a)** and locally refined map **(b).** The final resolution was estimated using the gold-standard FSC criterion at 0.143. Cryo-EM density maps are colored according to local resolution as calculated by CryoSPARC.

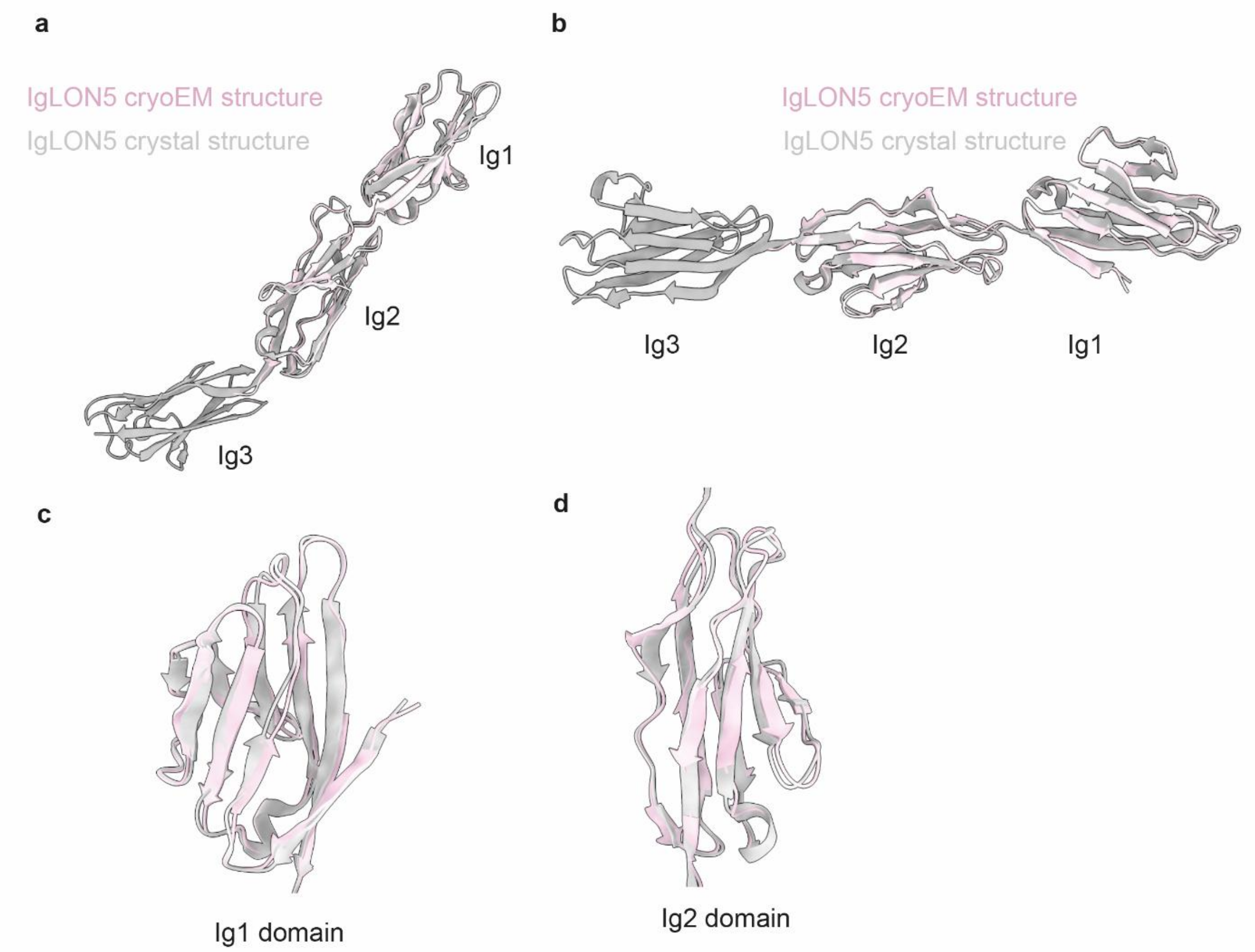

Supplementary Figure 8. Superimposed IgLON5 monomer. (a) Top view of the IgLON5 monomer crystal structure (PDB: 6DLE), shown in gray, overlaid with the cryo-EM structure of the IgLON5 monomer bound to Fab2513. (**b**) Side view of the overlay, illustrating the overall conservation of the IgLON5 monomer architecture in the presence and absence of Fab2513. **(c)** Overlay of the Ig1 domain from the crystal structure, shown in gray, and the cryo-EM structure, shown in pink. **(d)** Overlay of the Ig2 domain from the crystal structure, shown in gray, and the cryo-EM structure, shown in pink.

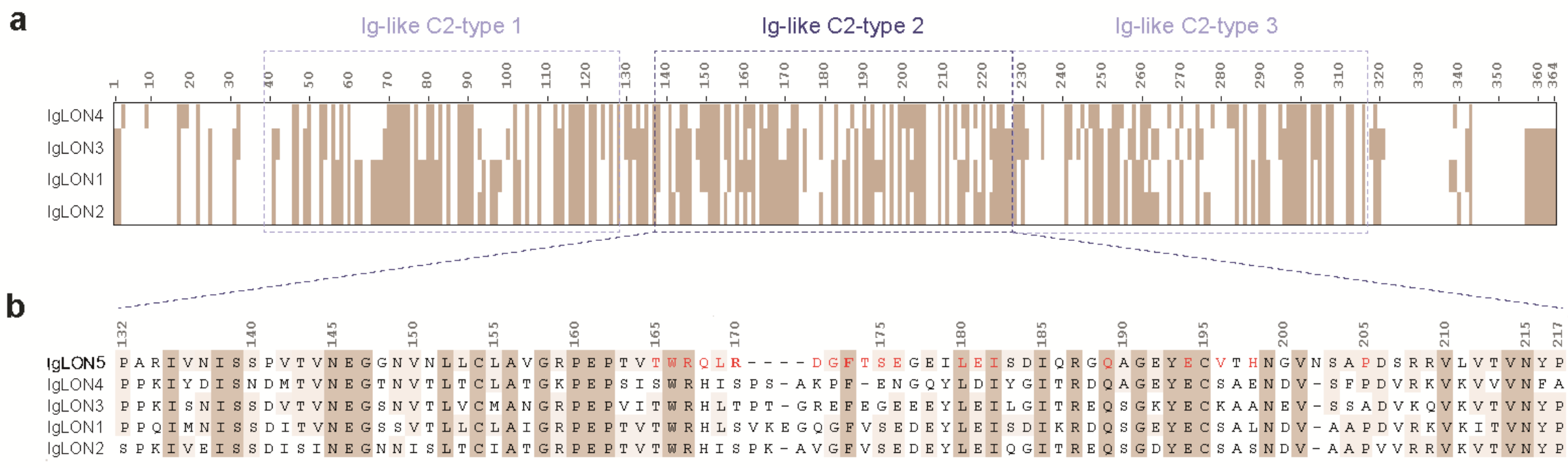

Supplementary Figure 9. **Sequence alignment of IgLON family proteins**. (**a**) Protein sequences were aligned using Clustal Omega. Proteins are ordered according to the Clustal Omega alignment. Amino acids identical to the reference protein, IgLON5, are highlighted in soft brown. Tick marks indicate every 10 amino acids. The three Ig-like C2-type domains are represented by dashed boxes. Top positions indicate alignment-relative numbering. (**b**) Alignment of the five IgLON family members in the Ig-like C2-type 2 domain. Residues conserved across all five proteins are highlighted in soft brown; lighter brown indicates residues conserved across two to four members of the IgLON family. Residues in contact with the heavy chain of mAb2513 are shown in red and are bolded if they also interact with the light chain. Top positions indicate IgLON5-relative numbering.

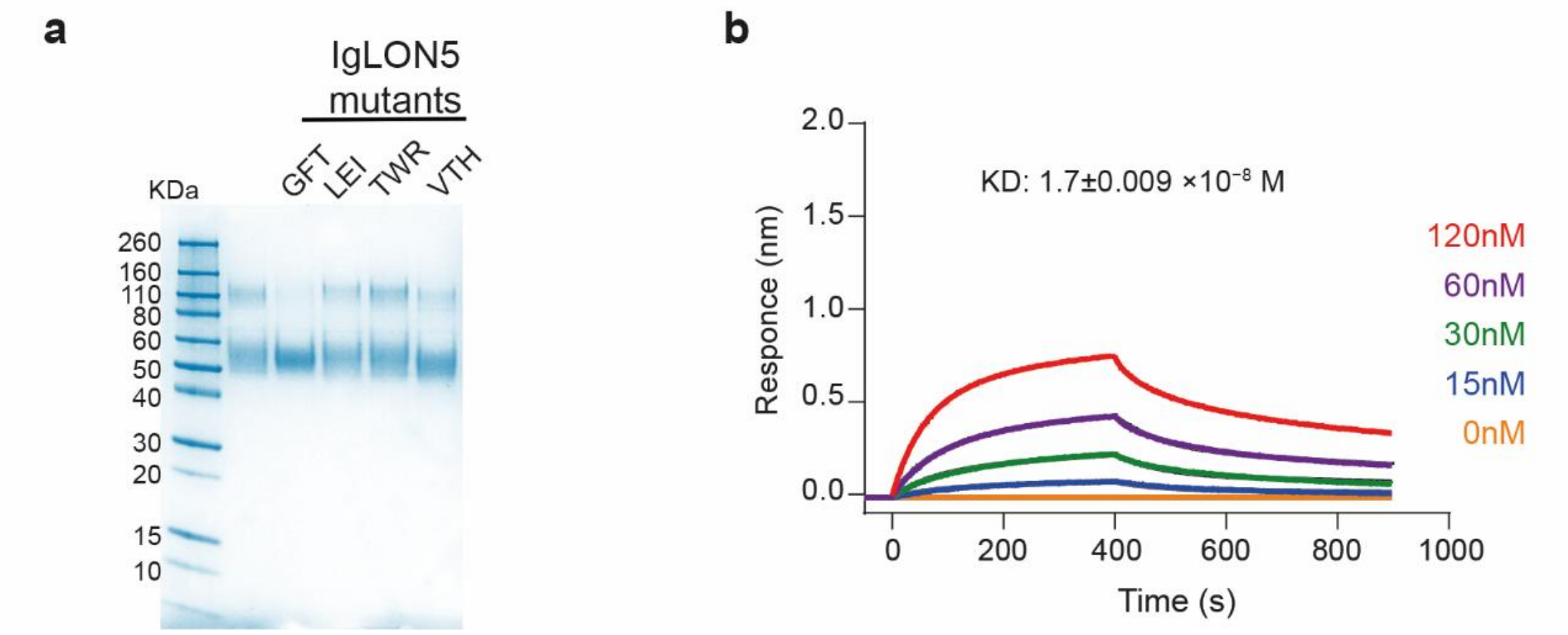

Supplementary Figure 10. Epitope validation by site-directed mutagenesis. (a) SDS–PAGE analysis under reducing conditions of purified IgLON5 mutant proteins. (b) Interaction of Fab2513 with the IgLON5 VLH mutant assessed by biolayer interferometry (BLI). Fab2513 was immobilized on FAB2G biosensors and exposed to increasing concentrations of the IgLON5 VLH mutant (15–120 nM). Binding responses (nm shift) were recorded. The sensorgram shown is representative of two independent experiments (n = 2)

>OPCMLΔC_His
MGVCGYLFLPWKCLVVVSLRLLFLVPTGVPVRSGDATFPKAMDNVTVRQGESATLRCTIDDRVTRVAWLNRSTILYA
GNDKWSIDPRVIILVNTPTQYSIMIQNVDVYDEGPYTCSVQTDNHPKTSRVHLIVQVPPQIMNISSDITVNEGSSVTLL
CLAIGRPEPTVTWRHLSVKEGQGFVSEDEYLEISDIKRDQSGEYECSALNDVAAPDVRKVKITVNYPPYISKAKNTG
VSVGQKGILSCEASAVPMAEFQWFKEETRLATGLDGMRIENKGRMSTLTFFNVSEKDYGNYTCVATNKLGNTNASIT
LYGPGAVIDGVNGSGSSAWSHPQFEKGGGSGGGGSGGSAWSHPQFEKGSGSGSGSGSHHHHHHHH
>NTRIΔC_His
MGVCGYLFLPWKCLVVVSLRLLFLVPTGVPVRSGDATFPKAMDNVTVRQGESATLRCTIDDRVTRVAWLNRSTILYA
GNDKWSIDPRVIILVNTPTQYSIMIQNVDVYDEGPYTCSVQTDNHPKTSRVHLIVQVPPQIMNISSDITVNEGSSVTLL
CLAIGRPEPTVTWRHLSVKEGQGFVSEDEYLEISDIKRDQSGEYECSALNDVAAPDVRKVKITVNYPPYISKAKNTG
VSVGQKGILSCEASAVPMAEFQWFKEETRLATGLDGMRIENKGRMSTLTFFNVSEKDYGNYTCVATNKLGNTNASIT
LYGPGAVIDGVNGSGSSAWSHPQFEKGGGSGGGGSGGSAWSHPQFEKGSGSGSGSGSHHHHHHHH
>LSAMPΔC_His
MVRRVQPDRKQLPLVLLRLLCLLPTGLPVRSVDFNRGTDNITVRQGDTAILRCVVEDKNSKVAWLNRSGIIFAGHDK
WSLDPRVELEKRHSLEYSLRIQKVDVYDEGSYTCSVQTQHEPKTSQVYLIVQVPPKISNISSDVTVNEGSNVTLVCM
ANGRPEPVITWRHLTPTGREFEGEEEYLEILGITREQSGKYECKAANEVSSADVKQVKVTVNYPPTITESKSNEATTG
RQASLKCEASAVPAPDFEWYRDDTRINSANGLEIKSTEGQSSLTVTNVTEEHYGNYTCVAANKLGVTNASLVLFRPG
SVRGINGSGSSAWSHPQFEKGGGSGGGGSGGSAWSHPQFEKGSGSGSGSGSHHHHHHHH
>NEGR1ΔC_His
MDMMLLVQGACCSNQWLAAVLLSLCCLLPSCLPAGQSVDFPWAAVDNMMVRKGDTAVLRCYLEDGASKGAWLN
RSSIIFAGGDKWSVDPRVSISTLNKRDYSLQIQNVDVTDDGPYTCSVQTQHTPRTMQVHLTVQVPPKIYDISNDMTV
NEGTNVTLTCLATGKPEPSISWRHISPSAKPFENGQYLDIYGITRDQAGEYECSAENDVSFPDVRKVKVVVNFAPTIQ
EIKSGTVTPGRSGLIRCEGAGVPPPAFEWYKGEKKLFNGQQGIIIQNFSTRSILTVTNVTQEHFGNYTCVAANKLGTTN
ASLPLNPPSTAQYGITGGSGSSAWSHPQFEKGGGSGGGGSGGSAWSHPQFEKGSGSGSGSGSHHHHHHHH
>NEGR1FL_Flag
MDMMLLVQGACCSNQWLAAVLLSLCCLLPSCLPAGQSVDFPWAAVDNMMVRKGDTAVLRCYLEDGASKGAWLN
RSSIIFAGGDKWSVDPRVSISTLNKRDYSLQIQNVDVTDDGPYTCSVQTQHTPRTMQVHLTVQVPPKIYDISNDMTV
NEGTNVTLTCLATGKPEPSISWRHISPSAKPFENGQYLDIYGITRDQAGEYECSAENDVSFPDVRKVKVVVNFAPTIQ
EIKSGTVTPGRSGLIRCEGAGVPPPAFEWYKGEKKLFNGQQGIIIQNFSTRSILTVTNVTQEHFGNYTCVAANKLGTTN
ASLPLNPPSTAQYGITGSADVLFSCWYLVLTLSSFTSIFYLKNAILQDYKDDDDK
> IgLON5ΔC_His
MPPPAPGARLRLLAAAALAGLAVISRGLLSQSLEFNSPADNYTVCEGDNATLSCFIDEHVTRVAWLNRSNILYAGNDR
WTSDPRVRLLINTPEEFSILITEVGLGDEGLYTCSFQTRHQPYTTQVYLIVHVPARIVNISSPVTVNEGGNVNLLCLAV
GRPEPTVTWRQLRDGFTSEGEILEISDIQRGQAGEYECVTHNGVNSAPDSRRVLVTVNYPPTITDVTSARTALGRAAL
LRCEAMAVPPADFQWYKDDRLLSSGTAEGLKVQTERTRSMLLFANVSARHYGNYTCRAANRLGASSASMRLLRPG
SLENSALEVLFQGPGGGSHHHHHHHHSAWSHPQFEKGGGSGGGGSGGSAWSHPQFEK
> IgLON5ΔC_Fc
MPPPAPGARLRLLAAAALAGLAVISRGLLSQSLEFNSPADNYTVCEGDNATLSCFIDEHVTRVAWLNRSNILYAGNDR
WTSDPRVRLLINTPEEFSILITEVGLGDEGLYTCSFQTRHQPYTTQVYLIVHVPARIVNISSPVTVNEGGNVNLLCLAV
GRPEPTVTWRQLRDGFTSEGEILEISDIQRGQAGEYECVTHNGVNSAPDSRRVLVTVNYPPTITDVTSARTALGRAAL
LRCEAMAVPPADFQWYKDDRLLSSGTAEGLKVQTERTRSMLLFANVSARHYGNYTCRAANRLGASSASMRLLRPG
SLENSAEPKSCDKTHTCPPCPAPELLGGPSVFLFPPKPKDTLMISRTPEVTCVVVDVSHEDPEVKFNWYVDGVEVHN
AKTKPREEQYNSTYRVVSVLTVLHQDWLNGKEYKCKVSNKALPAPIEKTISKAKGQPREPQVYTLPPSREEMTKNQ
VSLTCLVKGFYPSDIAVEWESNGQPENNYKTTPPVLDSDGSFFLYSKLTVDKSRWQQGNVFSCSVMHEALHNHYTQ
KSLSLSPGK
> IgLON5FL_Myc
MPPPAPGARLRLLAAAALAGLAVISRGLLSQSLEFNSPADNYTVCEGDNATLSCFIDEHVTRVAWLNRSNILYAGNDR
WTSDPRVRLLINTPEEFSILITEVGLGDEGLYTCSFQTRHQPYTTQVYLIVHVPARIVNISSPVTVNEGGNVNLLCLAV
GRPEPTVTWRQLRDGFTSEGEILEISDIQRGQAGEYECVTHNGVNSAPDSRRVLVTVNYPPTITDVTSARTALGRAAL
LRCEAMAVPPADFQWYKDDRLLSSGTAEGLKVQTERTRSMLLFANVSARHYGNYTCRAANRLGASSASMRLLRPG
SLENSAPRPPGLLALLSALGWLWWRMEQKLISEEDL

Supplementary Figure 11. Sequences of produced proteins. ΔC_His: constructs truncated at the ω-site and tagged with a C-terminal His$_6$ and tandem Strep-tag. FL: full-length constructs bearing either a Myc or FLAG tag. ΔC_Fc: constructs truncated at the ω-site and fused to a human IgG1 Fc domain.
