# Supplementary material for: Structural basis of IgLON5 autoantibody recognition in autoimmune encephalitis
Source: Nat Commun. 2026 Sep 11;17:10734. doi: 10.1038/s41467-026-77812-6 (PMC13658817; doi:10.1038/s41467-026-77812-6)
Supplement: Supplementary file 2 [file 41467_2026_77812_MOESM2_ESM.pdf]

nature portfolio

Peer Review File

Structural basis of IgLON5 autoantibody recognition in autoimmune encephalitis

Corresponding Author: Professor Laurent Perez

This file contains all reviewer reports in order by version, followed by all author rebuttals in order by version.

Version 0:

Reviewer comments:

Reviewer #1

(Remarks to the Author)
Roux et al. obtained the H+L sequences of an IgG4-specific IgLON5 antibody from a patient. They produced the Fab of this antibody recombinantly and analysed details of antigen recognition. The features of IgLON5 are of particular interest due to the link between autoantibodies and neurodegeneration. Both IgG1 and IgG4 antibodies to IgLON5 have been observed, but the contribution of either IgG1 or IgG4 to the pathogenesis of this disease is unclear. Cloning an IgLON5-specific mAb is a significant advance and will provide a valuable tool for gaining insight into this disease. But the present version of the paper has the following deficits.
1) They did not analyse the impact of the IgG4 isotype on functional activity. A unique feature of IgG4 is its ability to rapidly exchange Fab arms in vivo, resulting in monovalent antibodies. Therefore, the monovalent versus bivalent recognition of their mAb to IgLON5 and its functional consequences should be analysed.
2) They claim that their structural data indicate that the interaction is predominantly mediated by germline-encoded residues. While this would be intriguing, a quantitative analysis of antigen recognition by mutated and unmutated antibodies is lacking.
3) Their assertion that their data demonstrate that IgLON5 is polyclonal (see the abstract and line 328) may be premature. In fact, I would guess that the repertoire is polyclonal, but this is not demonstrated by their data. To reach such a conclusion, they should provide sequences of multiple IgLON5-specific Abs, not just one. A comparison with the total IgG4 repertoire, as they did, does not allow this conclusion.
4) I do not understand the basis of their statement in lines 399–400.

Reviewer #2

(Remarks to the Author)
In this study, the authors have isolated a high-affinity human IgG4 monoclonal antibody against Ig2 of IgLON5 and used cryo-EM to solve the structure of the complex. The structural and biochemical data show that the antibody does not block IgLON5's normal dimerization interfaces but instead induces clustering of IgLON5 dimers. This mechanism offers insight into how autoantibodies may drive neurodegeneration in anti-IgLON5 disease. Overall, this is a very interesting paper, the quality of the data is high, the relevance is very high as anti-IgLON5 diseases are recognized as very serious. There are few shortcomings highlighted below.

Line 86-88, references 13 and 14: I suggest "These trans interactions (across opposing membranes) generate a V-shaped complex compatible with synaptic cleft dimensions (13) and they likely contribute to the organization of chemical synapses (14)".
As far as I can tell, Ref. 14 (Mialon et al) ZIG-8 directly binds an acetylcholine receptor known as ACR-16 in C elegans, which does contribute to the organization of chemical synapse. But it is in Ref. 13 (Ranaivoison et al) that the V-shaped structure is shown for the first time and in the Graphical Abstract is mentioned to be "compatible with synaptic cleft dimensions".

Abstract and figure 2e: "higher-order clustering of IgLON5 dimers", is an important enough result to appear in the abstract. However, this result rests only on a single technique (gMFI) which is an indirect measurement. I feel that either the statements of aggregation need to be tone down or other orthogonal technologies should be used to demonstrate this point

without any doubt.

Line 209: Remove comma after “we detected”.

Line 204: “Separation of the constructs showed that IgLON5 forms a dimer, as expected”. The dimer is not apparent by looking at the SEC trace alone, unless it is compared to a monomer or a MW reference is added either as trace or a set of arrowheads on top of the curves.

Figure 2D: this type of data it is better presented as b/w images for the individual colors and the color panels only for the merge images. This has two advantages: first, it is easier for the eye to see the depth of the b/w contrast rather than red and blue over a black background. Second, as colour-blindness is a common condition, red and blue can be impossible to see for some individual.

Line 229: Figure 2f is not mentioned anywhere.

Line 246 - 265: the description of the IgLON5 interface is confusing as all the residue numbers are off by 27: in other words, T34 shown in figure 3D is actually T61 in the Uniprot entry for IgLON5. In odd contrast, figure 4H has the correct residue numbering. Whether this comes from not numbering the leader peptide or by numbering an artificial leader peptide, numbers should be consistent with the WT protein entry.

Figure 3: There should be a panel with the overlay of the published crystal structures vs this new structure, for two reasons: 1) identify deviations due to the Fab interaction on the Ig-Ig hinges, and 2) compare the cryo-EM and crystallographic structures in case there is some artifact related to the technology used.

Figure 4 and chapter “Characterization of Fab paratope and epitope”: Owing to the interesting nature of this complex, the fact that antibody binding is specific to IgLON5, and the caliber of the paper, the author should use mutagenesis to disrupt these interactions and unequivocally confirm their nature. Using the information of the atomic coordinates shown in figure 4 and the BLI assay, they should demonstrate that the interaction is breakable with the appropriate mutations.

Reviewer #3

(Remarks to the Author)
The manuscript by Roux et al. describes the isolation and characterization of a patient-derived anti-IgLON5 monoclonal antibody. Anti-IgLON5 disease is a rare neurological disorder that occupies a unique position at the intersection of autoimmunity and neurodegeneration. Although anti-IgLON5 autoimmunity has been recognized for nearly a decade, individual patient-derived antibody clones have not previously been isolated and characterized. Prior studies demonstrated that patient-derived antibodies disrupt neuronal IgLON5 clusters and induce neurodegenerative changes; however, the structural basis of antibody recognition and pathogenicity has remained unclear because most investigations relied on polyclonal patient sera rather than monoclonal antibodies.
To address this gap, the authors analyzed the B-cell repertoire of a patient with anti-IgLON5 disease, isolated an IgLON5-specific monoclonal antibody, and determined its structure in complex with IgLON5 using cryo-EM. The 3.7 Å cryo-EM structure revealed that IgLON5 forms a stable head-to-head homodimer mediated by interactions involving its first immunoglobulin-like domain (Ig1). The antibody recognizes a conformational epitope within the second immunoglobulin-like domain (Ig2), spanning residues 163–205. Structural analysis further showed that many key antigen-contact residues are germline-encoded, suggesting that autoreactive anti-IgLON5 antibodies may arise relatively readily from the naïve B-cell repertoire with limited affinity maturation.
The study also provides broader insights into the immunobiology of anti-IgLON5 disease. The findings suggest that the autoimmune response is unlikely to be driven by a single dominant pathogenic clone and instead may arise from a heterogeneous repertoire of autoreactive B cells. The identification of an IGHV4-39-derived antibody is particularly noteworthy, as this germline gene has been implicated in several other autoimmune disorders, suggesting that intrinsic features of specific antibody germlines predispose to self-reactivity. Furthermore, the substantial contribution of germline-encoded residues to antigen recognition suggests that only modest affinity maturation may be required to generate disease-associated antibodies. This observation is reminiscent of recent findings in anti-NMDAR encephalitis, where germline-encoded B-cell receptors can recognize NMDA receptors and limited affinity maturation is sufficient to generate pathogenic antibodies.
Overall, this is a carefully executed and highly significant study that represents a major advance in the field. To my knowledge, this is the first report describing the isolation of a patient-derived anti-IgLON5 monoclonal antibody and the structural characterization of its interaction with IgLON5. The work provides important mechanistic insights into both disease pathogenesis and the origins of autoreactive antibody responses. I fully support the publication of this manuscript and have only a few minor comments and suggestions.

Minor comments
1. Potential IgG–IgLON5 stoichiometry
The structural work focuses on the Fab–IgLON5 interaction, but it would be interesting to consider the potential stoichiometry of full-length IgG4 binding to dimeric IgLON5. Depending on the geometry of the epitope and receptor arrangement, multiple

binding configurations may be possible (e.g., IgG4:IgLON5 ratios of 1:2 or 2:2). This question is analogous to recent studies of anti-NMDAR autoantibodies, where Michalski et al. (NSMB, 2024) demonstrated that receptor–antibody stoichiometry is influenced by epitope location and can have important functional consequences, including differential effects on receptor function versus receptor internalization. While such experiments are clearly beyond the scope of the current work, a brief discussion of possible binding stoichiometries and their potential physiological implications could be valuable.

2. Role of germline reversion
The observation that many of the IgLON5-contacting residues in Fab2513 are germline encoded is particularly intriguing. It would be interesting in future studies to generate a germline-reverted version of Fab2513 and evaluate its affinity and specificity for IgLON5. Such experiments could directly test the authors' hypothesis regarding the limited affinity maturation required for antigen recognition. It would also help clarify whether somatic mutations primarily contribute through direct antigen contact or by modulating CDR conformations, structural flexibility, or binding dynamics. Although this question is outside the scope of the present manuscript, I'd be interested in knowing this information.

Version 1:

Reviewer comments:

Reviewer #1

(Remarks to the Author)
My points have been addressed in the revised version.

Reviewer #2

(Remarks to the Author)
The authors have revised the manuscript to a high standard and I have no further concerns.

Reviewer #3

(Remarks to the Author)
The authors addressed all of my minor suggestions as well as those of other reviewers. This is an important piece of work. Congratulations on your achievement.

Open Access This Peer Review File is licensed under a Creative Commons Attribution 4.0 International License, which permits use, sharing, adaptation, distribution and reproduction in any medium or format, as long as you give appropriate credit to the original author(s) and the source, provide a link to the Creative Commons license, and indicate if changes were

made.
In cases where reviewers are anonymous, credit should be given to 'Anonymous Referee' and the source.
The images or other third party material in this Peer Review File are included in the article’s Creative Commons license, unless indicated otherwise in a credit line to the material. If material is not included in the article’s Creative Commons license and your intended use is not permitted by statutory regulation or exceeds the permitted use, you will need to obtain permission directly from the copyright holder.
To view a copy of this license, visit https://creativecommons.org/licenses/by/4.0/

Responses to Reviewers

We thank the reviewers for their positive comments on our manuscript and for their constructive criticism, which helped us improve the manuscript. All additions and changes are highlighted in red in the revised version. Below, we provide a point-by-point response to the reviewers' comments. The reviewers' comments are shown in black and reproduced verbatim, while our responses are shown in blue.

Reviewer #1

Roux et al. obtained the H+L sequences of an IgG4-specific IgLON5 antibody from a patient. They produced the Fab of this antibody recombinantly and analysed details of antigen recognition. The features of IgLON5 are of particular interest due to the link between autoantibodies and neurodegeneration. Both IgG1 and IgG4 antibodies to IgLON5 have been observed, but the contribution of either IgG1 or IgG4 to the pathogenesis of this disease is unclear. Cloning an IgLON5-specific mAb is a significant advance and will provide a valuable tool for gaining insight into this disease. But the present version of the paper has the following deficits.

We thank the reviewer for finding our study of interest and for recognizing its significance.

1) They did not analyse the impact of the IgG4 isotype on functional activity. A unique feature of IgG4 is its ability to rapidly exchange Fab arms in vivo, resulting in monovalent antibodies. Therefore, the monovalent versus bivalent recognition of their mAb to IgLON5 and its functional consequences should be analysed.

Response: We agree that the functional consequences of the IgG4 isotype and the distinction between monovalent and bivalent binding are important points. To address this issue, we generated mAb2513 in its native IgG4 isotype and compared its activity with that of the corresponding IgG1 and monovalent Fab formats in our cell-based cross-linking assay. Both the IgG1 and IgG4 forms of mAb2513 increased the association of soluble fluorescently labelled IgLON5 with IgLON4- or IgLON5-expressing cells, although the effect of the IgG4 antibody was weaker than that of IgG1. In contrast, the monovalent Fab did not promote this association. These results indicate that monovalent binding is sufficient for antigen recognition, but that antibody bivalency is required to efficiently promote IgLON5 cross-linking or clustering at the cell surface (**Figure R1** and **Figure 2f** of the revised manuscript). These results indicate that mAb2513 retains the capacity to promote trans-association in its original IgG4 isotype, although with reduced efficacy, possibly because of the presence of functionally monovalent antibodies generated through Fab-arm exchange. Nevertheless, in a polyclonal setting containing additional IgG4 antibodies, Fab-arm exchange could further modify this effect and potentially reduce cross-linking even more, depending on the antigen specificity and epitope recognized by the exchanged Fab arm. We have now

acknowledged this limitation in the revised manuscript. Moreover, following Reviewer 2's suggestion, we have toned down our conclusions regarding antibody-mediated cross-linking throughout the manuscript.

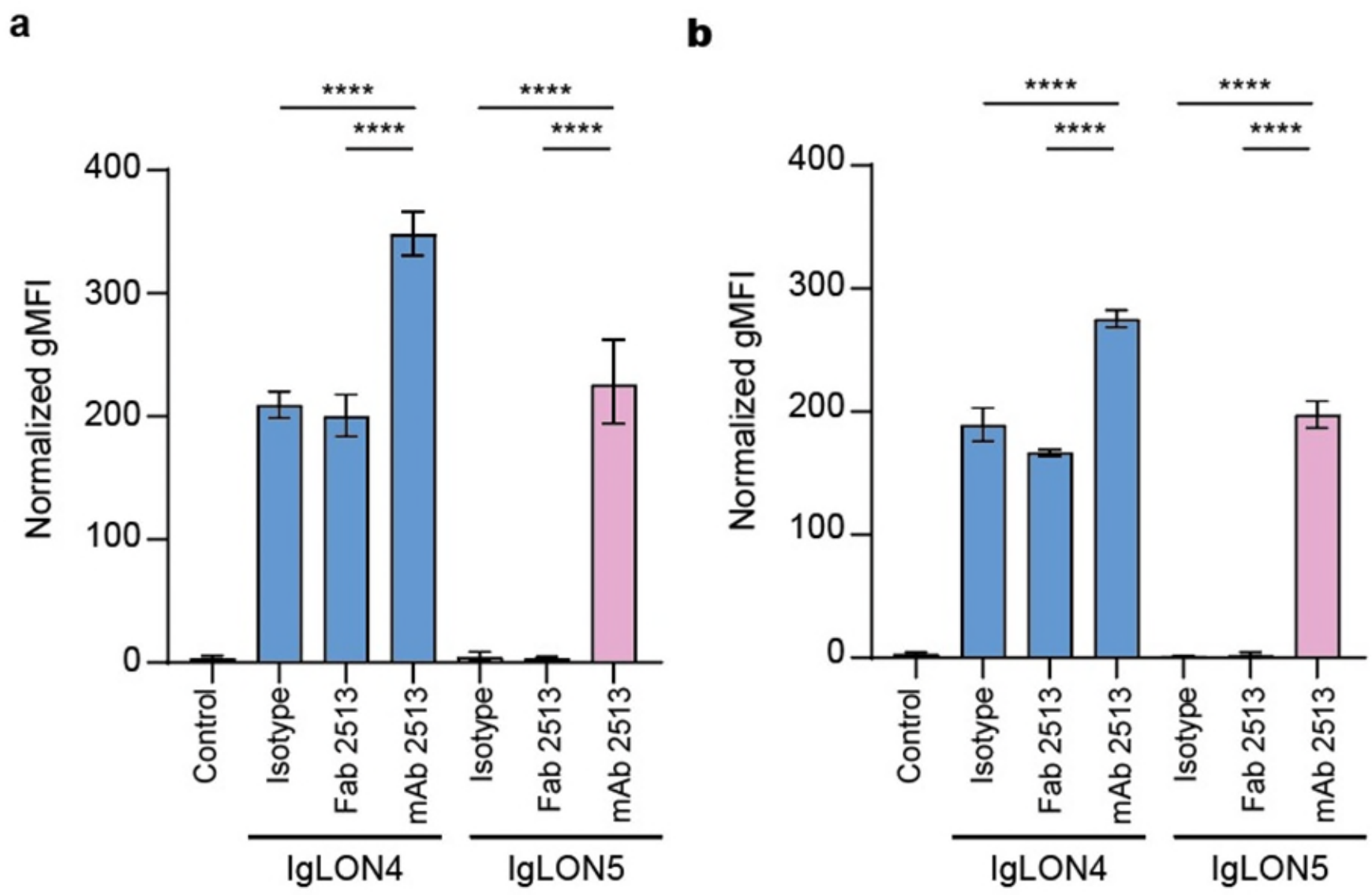

Figure R1. IgLON5 crosslinking with IgG4. Geometric mean fluorescence intensity of IgLON4- or IgLON5-transfected SH-SY5Y cells incubated with fluorescent soluble IgLON5 in the presence of an irrelevant antibody (isotype), mAb2513 as IgG1 (**a**) or IgG4 isotype (**b**), or Fab2513. Controls are mock transfected cells incubated with fluorescent soluble IgLON5. Data are presented as normalized geometric mean ± SD from three independent experiments (n = 3). *** $p < 0.005$, **** $p < 0.001$ (one-way ANOVA with Tukey's multiple comparisons test).

2) They claim that their structural data indicate that the interaction is predominantly mediated by germline-encoded residues. While this would be intriguing, a quantitative analysis of antigen recognition by mutated and unmutated antibodies is lacking.

Response: We thank the reviewer for pointing out this important experiment, which was missing from the previous version of the manuscript. To address this point, we inferred the unmutated common ancestor (UCA) of mAb2513 using ARPP UA Inference (Kepler 2013). Of note, the CDR3 sequences of the inferred UCA are identical to those of the mature mAb2513, whereas the somatic mutations present in the V and J genes were reverted to their germline sequences. We then performed BLI using the UCA as a Fab to quantify its affinity for IgLON5 (**Figure R2** and **Figure 4j** in the revised manuscript). As expected, the UCA retained detectable binding to IgLON5, although with reduced affinity compared with the affinity-matured antibody. These results indicate that initial antigen recognition is mediated by germline-encoded residues together with the conserved CDR3, whereas

somatic mutations acquired during affinity maturation stabilize the interaction with the antigen, mainly by reducing the dissociation rate.

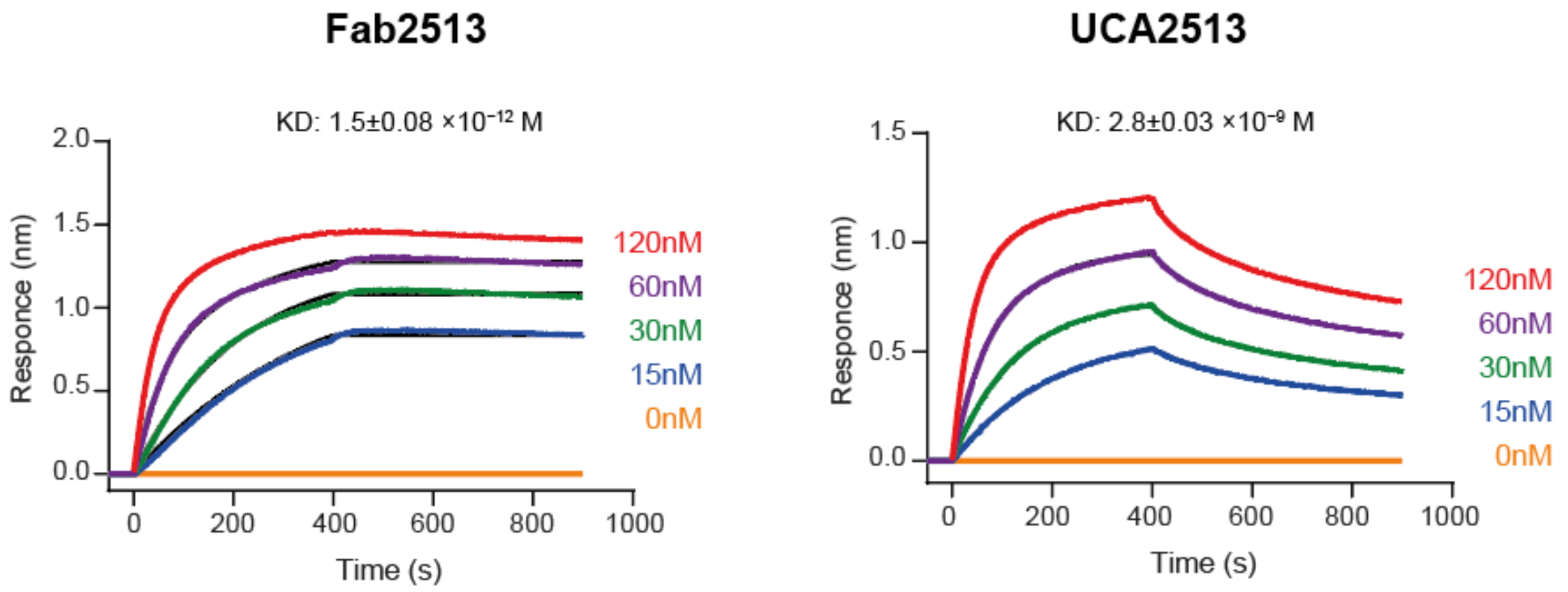

Figure R2. Measured affinity of Fab2513 and UCA on IgLON5. Affinity matured or UCA Fab-IgLON5 interaction assessed by biolayer interferometry (BLI). The Fab were immobilized on FAB2G biosensor, exposed to IgLON5 at increasing concentrations (15–120 nM) and binding responses (nm shift) were recorded.

3) Their assertion that their data demonstrate that IgLON5 is polyclonal (see the abstract and line 328) may be premature. In fact, I would guess that the repertoire is polyclonal, but this is not demonstrated by their data. To reach such a conclusion, they should provide sequences of multiple IgLON5-specific Abs, not just one. A comparison with the total IgG4 repertoire, as they did, does not allow this conclusion.

Response: We agree with the reviewer that our data do not formally demonstrate that the anti-IgLON5 response is polyclonal, as this would require the identification and sequencing of several independent IgLON5-specific mAbs, whereas we obtained only one. Although we generated more than 30 mAbs from the patient with anti-IgLON5 disease, only one was confirmed to be genuinely IgLON5-specific, and no additional autoreactive clones were identified. We nevertheless consider polyclonality to be a plausible working hypothesis. However, as requested by the reviewer, we have toned down our claims and now clearly present this interpretation as a hypothesis rather than a formally demonstrated conclusion.

4) I do not understand the basis of their statement in lines 399–400.

Response: We have revised the sentence to improve clarity.

Reviewer #2

In this study, the authors have isolated a high-affinity human IgG4 monoclonal antibody against Ig2 of IgLON5 and used cryo-EM to solve the structure of the complex. The structural and biochemical data show that the antibody does not block IgLON5's normal dimerization interfaces but instead induces clustering of IgLON5 dimers. This mechanism offers insight into how autoantibodies may drive neurodegeneration in anti-IgLON5 disease. Overall, this is a very interesting paper, the quality of the data is high, the relevance is very high as anti-IgLON5 diseases are recognized as very serious. There are few shortcomings highlighted below.

We thank the reviewer for finding our study interesting and of high quality.

Line 86-88, references 13 and 14: I suggest "These trans interactions (across opposing membranes) generate a V-shaped complex compatible with synaptic cleft dimensions (13) and they likely contribute to the organization of chemical synapses (14)". As far as I can tell, Ref. 14 (Mialon et al) ZIG-8 directly binds an acetylcholine receptor known as ACR-16 in C elegans, which does contribute to the organization of chemical synapse. But it is in Ref. 13 (Ranaivoison et al) that the V-shaped structure is shown for the first time and in the Graphical Abstract is mentioned to be "compatible with synaptic cleft dimensions".

Response: We thank the reviewer for identifying this referencing error. We have revised the text accordingly and corrected the relevant references. The revised text now reads as follows:

"IgLON dimerization is mediated by the first Ig domains, with the two protomers associating in a "head-to-head" configuration (Venkannagari et al. 2020). These *trans* interactions (across opposing membranes) generate a V-shaped complex compatible with synaptic cleft dimensions (Ranaivoson et al. 2019) and likely contribute to the organization of chemical synapses (Hashimoto, Maekawa, and Miyata 2009). Moreover, a study in *C. elegans* provided evidence that trans-synaptic IgLON interactions can organize neurochemical synapses and suggested that IgLONs may also interact with ionotropic receptors in the mammalian nervous system (Mialon et al. 2026)."

Abstract and figure 2e: "higher-order clustering of IgLON5 dimers", is an important enough result to appear in the abstract. However, this result rests only on a single technique (gMFI) which is an indirect measurement. I feel that either the statements of aggregation need to be tone down or other orthogonal technologies should be used to demonstrate this point without any doubt.

Response: We agree with the reviewer and have revised the manuscript to tone down this claim.

Line 209: Remove comma after "we detected".

Response: We edited the text accordingly.

Line 204: “Separation of the constructs showed that IgLON5 forms a dimer, as expected”. The dimer is not apparent by looking at the SEC trace alone, unless it is compared to a monomer or a MW reference is added either as trace or a set of arrowheads on top of the curves.

Response: We thank the reviewer for pointing out this omission, arrowheads representing the elution volume of thyroglobulin (669 kDa), ferritin (440 kDa), BSA (66 kDa), and myoglobin (17 kDa) were added to the panel.

Figure 2D: this type of data it is better presented as b/w images for the individual colors and the color panels only for the merge images. This has two advantages: first, it is easier for the eye to see the depth of the b/w contrast rather than red and blue over a black background. Second, as colour-blindness is a common condition, red and blue can be impossible to see for some individual.

Response: We agree with the reviewer’s suggestion. The individual images are now displayed in black and white, while the merged images are shown in color.

Line 229: Figure 2f is not mentioned anywhere.

Response: We apologize for this mistake. It has been corrected in the revised manuscript.

Line 246 - 265: the description of the IgLON5 interface is confusing as all the residue numbers are off by 27: in other words, T34 shown in figure 3D is actually T61 in the Uniprot entry for IgLON5. In odd contrast, figure 4H has the correct residue numbering. Whether this comes from not numbering the leader peptide or by numbering an artificial leader peptide, numbers should be consistent with the WT protein entry.

Response: We thank the reviewer for pointing out this mistake. Indeed, one of the models was built using residue numbering starting after the signal peptide. This has been corrected throughout the revised manuscript.

Figure 3: There should be a panel with the overlay of the published crystal structures vs this new structure, for two reasons: 1) identify deviations due to the Fab interaction on the Ig-Ig hinges, and 2) compare the cryo-EM and crystallographic structures in case there is some artifact related to the technology used.

Response: We thank the reviewer for pointing out this omission. We have now superimposed the two structures, as shown in **Figure R3** and in the new Supplementary Figure 8 of the revised manuscript. To complement this visual comparison, we performed a pairwise structural alignment using TM-align (Bittrich et al. 2024). This analysis showed that the Fab-bound IgLON5 dimer closely matches the previously published crystal structure of the IgLON5 dimer, with an RMSD of 1.4 Å, indicating that the overall dimeric organization is preserved upon Fab binding. This point has also been added to the revised manuscript.

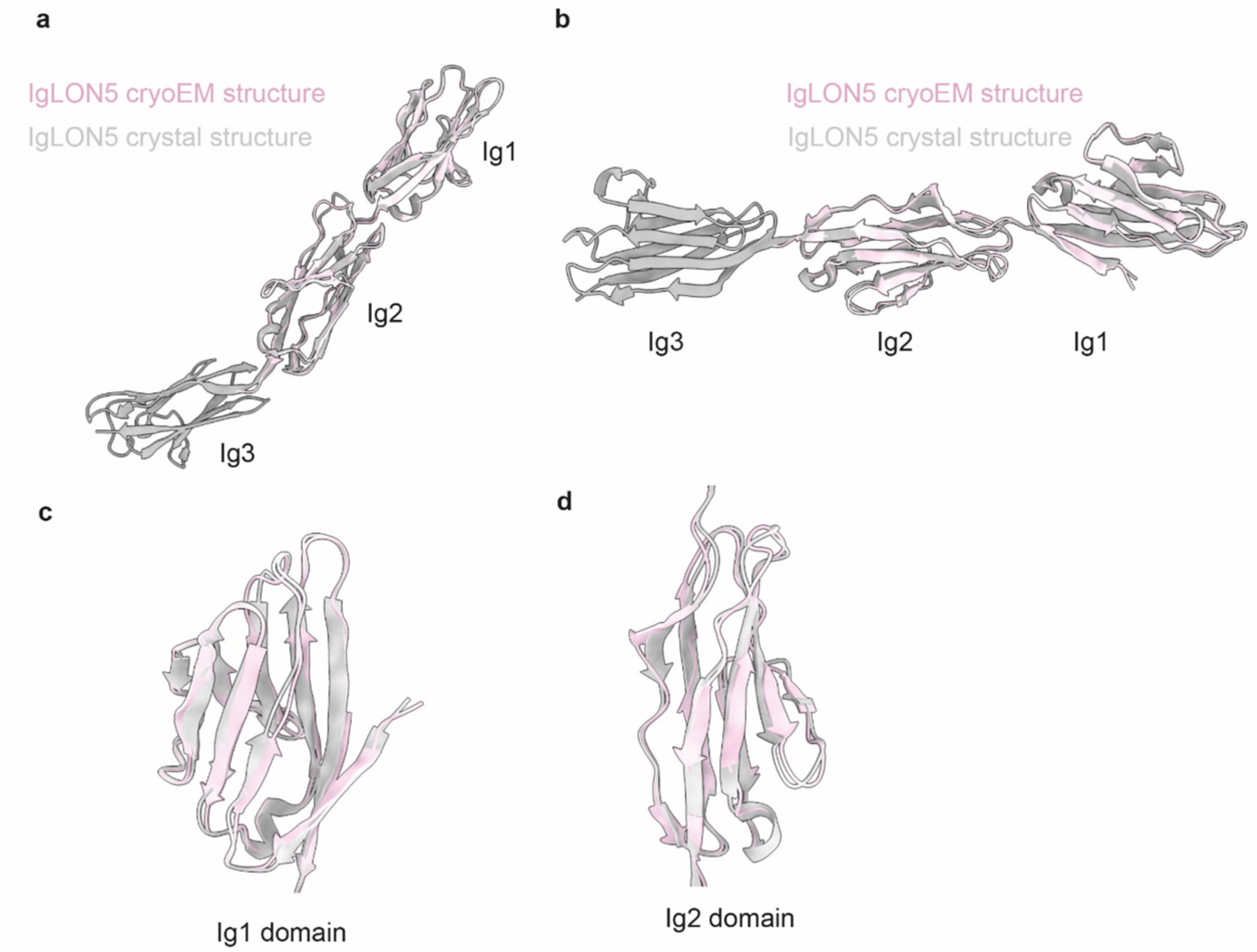

Figure R3. Superimposed IgLON5 monomer. (a) Top view of the IgLON5 crystal structure (PDB: 6DLE), shown in gray, overlaid with the cryo-EM structure of the IgLON5 monomer bound to Fab2513. **(b)** Side view of the overlay, illustrating the overall conservation of the IgLON5 monomer architecture in the presence and absence of Fab2513. **(c)** Overlay of the Ig1 domain from the crystal structure, shown in gray, and the cryo-EM structure, shown in pink. **(d)** Overlay of the Ig2 domain from the crystal structure, shown in gray, and the cryo-EM structure, shown in pink.

Figure 4 and chapter "Characterization of Fab paratope and epitope": Owing to the interesting nature of this complex, the fact that antibody binding is specific to IgLON5, and the caliber of the paper, the author should use mutagenesis to disrupt these interactions and unequivocally confirm their nature. Using the information of the atomic coordinates shown in figure 4 and the BLI assay, they should demonstrate that the interaction is breakable with the appropriate mutations.

Response: We thank the reviewer for pointing out this omission. To validate the residues identified by cryo-EM analysis, we generated several IgLON5 mutants in which four clusters of three amino acids were replaced by alanine: $_{165}$-TWR-$_{167}$, $_{172}$-GFT-$_{174}$, $_{180}$-LEI-$_{182}$, and $_{196}$-VTH-$_{198}$. We then evaluated Fab2513 binding by BLI and confirmed a complete loss of binding for the TWR, GFT, and LEI mutants (**Fig. R4a** and **Fig. 4i** of the revised manuscript).

For the VTH mutant, residual binding was still observed. We therefore quantified Fab2513 binding to this mutant and found a K_D of $1.7 \pm 0.009 \times 10^{-8}$ M, compared with $1.5 \pm 0.08 \times 10^{-12}$ M for the wild-type protein (**Fig. R4b** and **Supplementary Fig. 10** of the revised manuscript). This indicates that the VTH cluster contributes to Fab2513 binding, but that its mutation results in an intermediate phenotype, with strongly reduced but still detectable binding. We explain this residual interaction by the fact that, although V196 and H198 contact CDRH2 residues Y52 and F53 of Fab2513, these contacts are embedded within a larger interlocked and shape-complementary interface that is not fully disrupted by the VTH-to-alanine mutation.

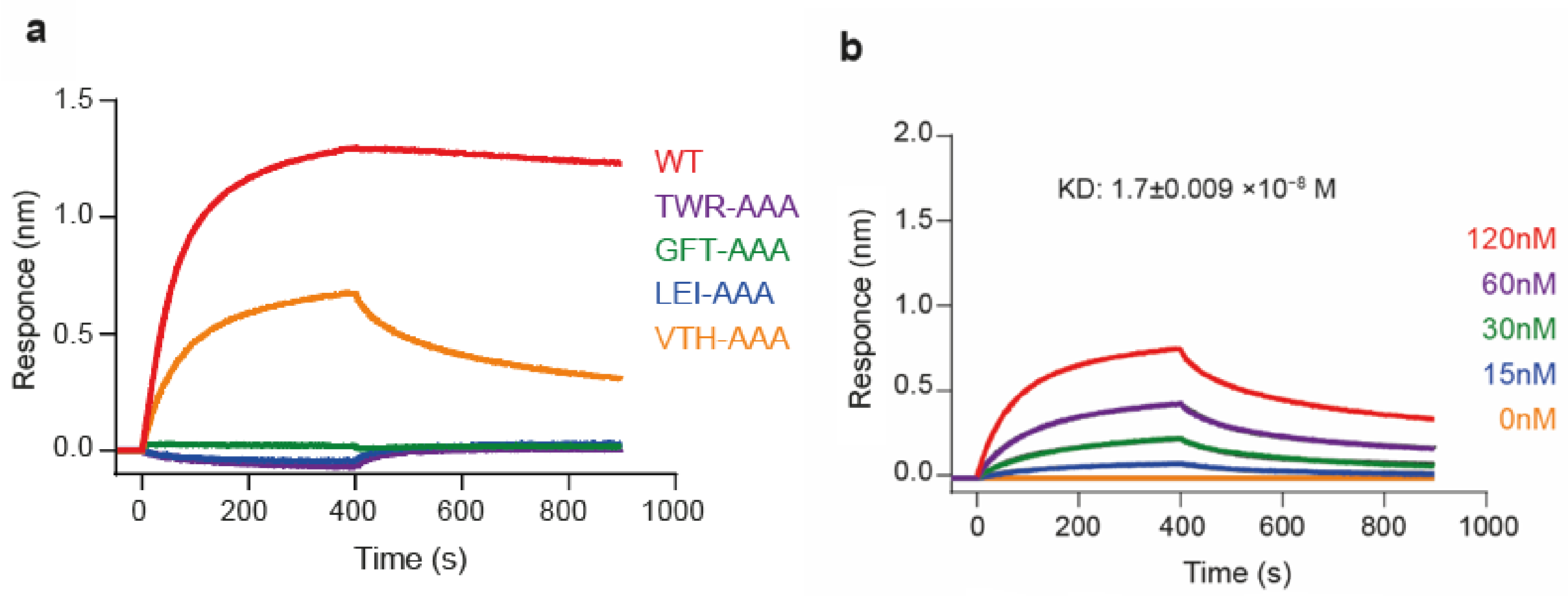

Fig. R4. Alanine-cluster mutations disrupt mAb2513 interaction with IgLON5. (a) Fab–IgLON5 interaction measured for the wild-type protein (red) and alanine-cluster mutants: TWR (violet), GFT (green), LEI (blue), and VTH (orange). **(b)** Binding kinetics of Fab2513 interaction with the IgLON5 VTH mutant, assessed by BLI.

Reviewer #3

The manuscript by Roux et al. describes the isolation and characterization of a patient-derived anti-IgLON5 monoclonal antibody. Anti-IgLON5 disease is a rare neurological disorder that occupies a unique position at the intersection of autoimmunity and neurodegeneration. Although anti-IgLON5 autoimmunity has been recognized for nearly a decade, individual patient-derived antibody clones have not previously been isolated and characterized. Prior studies demonstrated that patient-derived antibodies disrupt neuronal IgLON5 clusters and induce neurodegenerative changes; however, the

structural basis of antibody recognition and pathogenicity has remained unclear because most investigations relied on polyclonal patient sera rather than monoclonal antibodies.

To address this gap, the authors analyzed the B-cell repertoire of a patient with anti-IgLON5 disease, isolated an IgLON5-specific monoclonal antibody, and determined its structure in complex with IgLON5 using cryo-EM. The 3.7 Å cryo-EM structure revealed that IgLON5 forms a stable head-to-head homodimer mediated by interactions involving its first immunoglobulin-like domain (Ig1). The antibody recognizes a conformational epitope within the second immunoglobulin-like domain (Ig2), spanning residues 163–205. Structural analysis further showed that many key antigen-contact residues are germline-encoded, suggesting that autoreactive anti-IgLON5 antibodies may arise relatively readily from the naïve B-cell repertoire with limited affinity maturation.

The study also provides broader insights into the immunobiology of anti-IgLON5 disease. The findings suggest that the autoimmune response is unlikely to be driven by a single dominant pathogenic clone and instead may arise from a heterogeneous repertoire of autoreactive B cells. The identification of an IGHV4-39-derived antibody is particularly noteworthy, as this germline gene has been implicated in several other autoimmune disorders, suggesting that intrinsic features of specific antibody germlines predispose to self-reactivity. Furthermore, the substantial contribution of germline-encoded residues to antigen recognition suggests that only modest affinity maturation may be required to generate disease-associated antibodies. This observation is reminiscent of recent findings in anti-NMDAR encephalitis, where germline-encoded B-cell receptors can recognize NMDA receptors and limited affinity maturation is sufficient to generate pathogenic antibodies.

Overall, this is a carefully executed and highly significant study that represents a major advance in the field. To my knowledge, this is the first report describing the isolation of a patient-derived anti-IgLON5 monoclonal antibody and the structural characterization of its interaction with IgLON5. The work provides important mechanistic insights into both disease pathogenesis and the origins of autoreactive antibody responses. I fully support the publication of this manuscript and have only a few minor comments and suggestions.

We thank the reviewer for finding our work well executed and of interest.

Minor comments

1. Potential IgG–IgLON5 stoichiometry

The structural work focuses on the Fab–IgLON5 interaction, but it would be interesting to consider the potential stoichiometry of full-length IgG4 binding to dimeric IgLON5. Depending on the geometry of the epitope and receptor arrangement, multiple binding configurations may be possible (e.g., IgG4:IgLON5 ratios of 1:2 or 2:2). This question is analogous to recent studies of anti-NMDAR autoantibodies, where Michalski et al. (NSMB, 2024) demonstrated that receptor–antibody stoichiometry is influenced by epitope location and can have important functional consequences,

including differential effects on receptor function versus receptor internalization. While such experiments are clearly beyond the scope of the current work, a brief discussion of possible binding stoichiometries and their potential physiological implications could be valuable.

Response: We thank the reviewer for this insightful suggestion. We agree that the stoichiometry of full-length IgG4 binding to membrane-associated, dimeric IgLON5 is an important point to consider. Our structural data define the Fab–IgLON5 interface, but they do not directly establish the architecture of the full-length IgG/IgLON5 complex at the cell surface. Indeed, a single bivalent IgG molecule could engage two IgLON5 molecules between adjacent dimers, corresponding to an apparent 1:2 IgG complex. Alternatively, each IgLON5 molecule within a dimer could be occupied by a distinct IgG molecule, generating a 2:2 arrangement. These configurations would have different potential consequences: intradimer or interdimer cross-linking could stabilize IgLON5 assemblies or promote higher-order clustering, whereas monovalent or sterically constrained binding may primarily block or alter specific IgLON5 interactions without extensive receptor reorganization. We have added a short discussion of this point to the revised manuscript. In this paragraph, we also refer to the recent anti-NMDAR autoantibody studies by Michalski et al., which showed that epitope location can influence antibody–receptor stoichiometry and that distinct binding architectures may have different functional consequences, including effects on receptor function and synaptic localization/internalization. Our own data showing that full-length IgG4 mAb2513 can increase IgLON5 association (please see our response to the **Reviewer** 1), although less efficiently than the corresponding IgG1, are consistent with the idea that antibody valency and geometry may influence IgLON5 organization at the membrane. We now explicitly state that defining the stoichiometry of full-length IgG4–IgLON5 complexes will require dedicated experiments, which we think are beyond the scope of the present study.

The text can be read as follow:

Notably, our data demonstrate that Fab binding does not disrupt the IgLON5 dimerization interface but instead is compatible with, and may promote, higher-order homo- or hetero-multimerization. Such antibody-induced clustering has been reported to trigger receptor internalization and may contribute to neuronal dysfunction and tau pathology (Askin et al. 2025). In this context, an important question raised by our structural findings is the potential stoichiometry of full-length IgG4 binding to dimeric, membrane-associated IgLON5. Several configurations can be envisioned. One IgG molecule could bridge two IgLON5 protomers within a dimer or, perhaps more plausibly given the pre-existing dimeric arrangement and membrane constraints, engage IgLON5 molecules from neighbouring dimers, thereby promoting higher-order clustering. Alternatively, each protomer within an IgLON5 dimer could be bound by a distinct IgG molecule, resulting in a 2:2 IgG arrangement that may further stabilize or organize multimeric assemblies. These possibilities are particularly relevant for IgG4 antibodies, whose functional monovalency after Fab-arm exchange may reduce, but not necessarily

abolish, their capacity to cross-link membrane IgLON5. This is consistent with our observation that full-length mAb2513 in the IgG4 format enhances IgLON5 association less efficiently than the corresponding IgG1. Similar principles have recently been described for anti-NMDAR autoantibodies, where epitope location and antibody–receptor stoichiometry influence functional outcomes, including receptor modulation and internalization (Michalski et al. 2024). Future studies using full-length antibodies, membrane-tethered IgLON5, and quantitative imaging or structural approaches will be required to define the precise stoichiometry of IgG4–IgLON5 complexes and determine how this geometry contributes to IgLON5 clustering and downstream pathogenic effects.

2. Role of germline reversion

The observation that many of the IgLON5-contacting residues in Fab2513 are germline encoded is particularly intriguing. It would be interesting in future studies to generate a germline-reverted version of Fab2513 and evaluate its affinity and specificity for IgLON5. Such experiments could directly test the authors' hypothesis regarding the limited affinity maturation required for antigen recognition. It would also help clarify whether somatic mutations primarily contribute through direct antigen contact or by modulating CDR conformations, structural flexibility, or binding dynamics. Although this question is outside the scope of the present manuscript, I'd be interested in knowing this information.

Response: We thank the reviewer for pointing out this important experiment, which was also raised by Reviewer 1 and was missing from the previous version of the manuscript. Please see our response to Reviewer 1 for details. We have also added a short paragraph to the Discussion to highlight this point.

The text can be read as follow:

The paratope of mAb2513 involves all three CDRs of the heavy chain and CDR3 of the light chain, with several contacts mediated by residues located in CDRH1 and CDRH2. Because these regions are partly germline encoded, this binding mode suggested that the naïve precursor of mAb2513 might already possess intrinsic reactivity toward IgLON5. Consistent with this hypothesis, the inferred unmutated common ancestor of mAb2513, in which the mature CDR3 sequences are preserved while somatic mutations in the V and J genes are reverted to their germline configuration, retained detectable binding to IgLON5. This indicates that initial antigen recognition is supported by a combination of germline-encoded residues and the conserved CDR3 loops. However, the reduced affinity of the UCA compared with the mature antibody, mainly due to a faster dissociation rate, suggests that somatic mutations acquired during affinity maturation stabilize the interaction with IgLON5. These findings support a model in which low-affinity self-reactivity may be present at the precursor stage and subsequently strengthened through affinity maturation. This mechanism is reminiscent of recent observations in anti-NMDAR autoimmunity, where germline or near-germline BCRs with low-affinity self-reactivity have been implicated in the emergence of pathogenic antibodies in the context of human

breast cancer (Kleeman et al. 2026) and have also been described in several autoimmune encephalitides (Stöffler et al. 2026).

References

Askin, Bilge, Cagla Kilic, César Cordero Gómez, Sophie Lan-Linh Duong, Alvaro Domingues-Baquero, Alexander Goihl, Karsten Nalbach, Joana Petushi, Pia Grundschöttel, Jessica Wagner, Valentine Thomas, Janne Lamberty, Emily Withers, Hanna Huber, Sabrina Huebschmann, Ekaterina Semenova, Paul Turko, Andrew G. Newman, Lisa Diez, Marc Beyer, Elena De Domenico, Peter Körtvelyessy, Dirk Reinhold, Anja Schneider, Jonas J. Neher, Thomas Ulas, Stefan F. Lichtenthaler, Benjamin R. Rost, Dietmar Schmitz, Harald Prüss, and Susanne Wegmann. 2025. 'IgLON5 autoimmune antibodies activate Tau via neuronal hyperactivity', *bioRxiv*: 2024.03.10.584272.

Bittrich, Sebastian, Joan Segura, Jose M Duarte, Stephen K Burley, and Yana Rose. 2024. 'RCSB protein Data Bank: exploring protein 3D similarities via comprehensive structural alignments', *Bioinformatics*, 40.

Hashimoto, Takashi, Shohei Maekawa, and Seiji Miyata. 2009. 'IgLON cell adhesion molecules regulate synaptogenesis in hippocampal neurons', *Cell Biochemistry and Function*, 27: 496–98.

Kepler, T. B. 2013. 'Reconstructing a B-cell clonal lineage. I. Statistical inference of unobserved ancestors', *F1000Res*, 2: 103.

Kleeman, Sam O., Kevin Michalski, Xiang Zhao, Ruben Steigerwald, Miriam Ferrer, Llewelyn Levett, Ethan Ertel, Austin Schultz, Noriko Simorowski, Pamela Moody, Tse-Luen Wee, Cristina Valente, Sharon Fox, Mateusz Makuch, Selina Thomsen, Ruby Harrison, Claire Regan, Jonathan Preall, Qing Gao, Dennis Thomas, Jill Habel, Rachel Rubino, Sarosh Irani, Hiro Furukawa, and Tobias Janowitz. 2026. 'Ectopic NMDAR expression in cancer unmasks germline-encoded autoimmunity', *Nature*.

Mialon, Morgane, Liubov Patrash, Laure Granger, Weinreb Alexis, Engin Özkan, Jean-Louis Bessereau, and Berangere Pinan-Lucarre. 2026. 'A trans-synaptic IgLON adhesion molecular complex directly contacts and clusters a nicotinic receptor', *Nat Commun*, 17: 1404.

Michalski, K., T. Abdulla, S. Kleeman, L. Schmidl, R. Gómez, N. Simorowski, F. Vallese, H. Prüss, M. Heckmann, C. Geis, and H. Furukawa. 2024. 'Structural and functional mechanisms of anti-NMDAR autoimmune encephalitis', *Nat Struct Mol Biol*, 31: 1975–86.

Ranaivoson, F. M., L. S. Turk, S. Ozgul, S. Kakehi, S. von Daake, N. Lopez, L. Trobiani, A. De Jaco, N. Denissova, B. Demeler, E. Özkan, G. T. Montelione, and D. Comoletti. 2019. 'A Proteomic Screen of Neuronal Cell-Surface Molecules Reveals IgLONs as Structurally Conserved Interaction Modules at the Synapse', *Structure*, 27: 893–906.e9.

Stöffler, L., J. Kreye, F. A. Arlt, E. Sanchez-Sendin, J. Hoffmann, H. C. Kornau, M. Kirchner, P. Mertins, S. van Hoof, S. Wernick, M. Homeyer, S. M. Reincke, and H. Prüss. 2026. 'High-Affinity Human Germline and Mutated Autoantibodies Commonly Cross-React to Self-Antigens in 5 Subgroups of Autoimmune Encephalitis', *Neurol Neuroimmunol Neuroinflamm*, 13: e200608.

Venkannagari, Harikanth, James M. Kasper, Anurag Misra, Scott A. Rush, Shanghua Fan, Hubert Lee, Hong Sun, Suchithra Seshadrinathan, Mischa Machius, Jonathan D. Hommel, and Gabby Rudenko. 2020. 'Highly Conserved Molecular Features in IgLONs Contrast Their Distinct Structural and Biological Outcomes', *J Mol Biol*, 432: 5287–303.

Responses to Reviewers

We thank the reviewers for their positive comments on our manuscript and for their constructive criticism, which helped us improve the manuscript. Below, we provide a point-by-point response to the reviewers' comments. The reviewers' comments are shown in black and reproduced verbatim, while our responses are shown in blue.

Reviewer #1

My points have been addressed in the revised version.

We thank the reviewer for finding our study of interest and for recognizing its significance.

Reviewer #2

The authors have revised the manuscript to a high standard and I have no further concerns.

We thank the reviewer for their positive assessment of our work and for considering it to be of a high standard:

Reviewer #3

The authors addressed all of my minor suggestions as well as those of other reviewers. This is an important piece of work. Congratulations on your achievement.

We thank the reviewer for this insightful suggestion and for their positive and enthusiastic assessment of our manuscript.
